# Ironing Out the Unconventional Mechanisms of Iron Acquisition and Gene Regulation in *Chlamydia*

**DOI:** 10.3389/fcimb.2017.00394

**Published:** 2017-09-08

**Authors:** Nick D. Pokorzynski, Christopher C. Thompson, Rey A. Carabeo

**Affiliations:** ^1^School of Molecular Biosciences, College of Veterinary Medicine, Washington State University Pullman, WA, United States; ^2^Jefferiss Trust Laboratories, Faculty of Medicine, Imperial College London, St. Mary's Hospital London, United Kingdom

**Keywords:** ABC-type permease-repressor fusion, vesicular iron, intracellular pathogen, persistence, iron homeostasis

## Abstract

The obligate intracellular pathogen *Chlamydia trachomatis*, along with its close species relatives, is known to be strictly dependent upon the availability of iron. Deprivation of iron *in vitro* induces an aberrant morphological phenotype termed “persistence.” This persistent phenotype develops in response to various immunological and nutritional insults and may contribute to the development of sub-acute *Chlamydia*-associated chronic diseases in susceptible populations. Given the importance of iron to *Chlamydia*, relatively little is understood about its acquisition and its role in gene regulation in comparison to other iron-dependent bacteria. Analysis of the genome sequences of a variety of chlamydial species hinted at the involvement of unconventional mechanisms, being that *Chlamydia* lack many conventional systems of iron homeostasis that are highly conserved in other bacteria. Herein we detail past and current research regarding chlamydial iron biology in an attempt to provide context to the rapid progress of the field in recent years. We aim to highlight recent discoveries and innovations that illuminate the strategies involved in chlamydial iron homeostasis, including the vesicular mode of acquiring iron from the intracellular environment, and the identification of a putative iron-dependent transcriptional regulator that is synthesized as a fusion with a ABC-type transporter subunit. These recent findings, along with the noted absence of iron-related homologs, indicate that *Chlamydia* have evolved atypical approaches to the problem of iron homeostasis, reinvigorating research into the iron biology of this pathogen.

## Introduction

Being the fourth most abundant element in the Earth's crust, iron (Fe) has developed a rich evolutionary history with life on Earth (Frey and Reed, 2012). Iron is an essential micronutrient for nearly every organism with very few notable exceptions. One such exception is *Borrelia burgdorferi*, which is thought to substitute iron for other metals like manganese (Mn) in its metabolism (Posey and Gherardini, 2000). The requirement for iron is no less significant for the wide array of prokaryotic human pathogens, where the limitation of iron is generally lethal. This suggests biochemical intervention in iron-related pathways may be a promising medical avenue for the treatment of infectious disease. Pathogenic bacteria, like many other organisms, utilize iron in a wide array of conserved biochemical pathways, including prominent examples such as the tricarboxcylic acid (TCA) cycle and the electron transport chain (Oexle et al., 1999; Kim et al., 2012). However, in all organisms, iron dependency comes with significant problems that must be overcome. Biologically available iron is commonly in one of either two oxidative states: ferric (Fe^3+^) or ferrous (Fe^2+^) iron. Ferric iron is notorious for its insolubility at physiological pH, and therefore its biological utility is restricted unless reduced to Fe^2+^. Moreover, free Fe^2+^ can react with hydrogen peroxide in the Fenton reaction and generate hydroxyl radicals associated with cellular oxidative stress (e.g., DNA damage; Winterbourn, 1995). Indeed, perturbation of iron-dependent regulons has been demonstrated to facilitate the acquisition of antimicrobial resistance in *E. coli*, likely by dysregulating iron acquisition and promoting the accumulation of free iron that can participate in DNA mutagenesis (Mehi et al., 2014). Accordingly, iron-dependent organisms are presented with a tripartite challenge of acquiring iron that is in the appropriate oxidative state, in homeostatic amounts, and properly liganded to avoid collateral damage to the cell. For bacterial pathogens, this is complicated further by the important task of acquiring iron *from the host*. As such, pathogenic bacteria have evolved an impressive array of iron acquisition systems and iron-responsive regulatory networks to enhance their success when competing for iron. Iron acquisition and homeostasis have been extensively researched for many important prokaryotic organisms including *Escherichia coli, Salmonella typhimurium*, and *Mycobacterium tuberculosis* (Andrews et al., 2003). It is well-documented in the literature that the *Chlamydiaceae* are strictly iron-dependent (Raulston, 1997; Al-Younes et al., 2001; Freidank et al., 2001), but the long-standing genetic intractability of the organism has rendered many benchmark approaches to iron biology ineffectual in past chlamydial research. Consequently, our understanding of this fundamental biological process is incomplete in Chlamydiae.

The *Chlamydiaceae* includes many pathogens of humans and economically important animals, including *Chlamydia trachomatis* (genital and ocular human infections), *Chlamydia pneumoniae* (pulmonary human infection), *Chlamydia pecorum* (cattle/sheep) and *Chlamydia suis* (swine). Chlamydiae are Gram-negative obligate intracellular pathogens typified by a unique biphasic developmental cycle that interconverts an infectious and metabolically dormant elementary body (EB) to a vegetative and metabolically active reticulate body (RB) (AbdelRahman and Belland, 2005). Interestingly, Chlamydiae can deviate from their normal developmental cycle under stress conditions, resulting in the formation of aberrantly enlarged RB forms that fail to divide or differentiate and are distinguished by dysregulated gene expression (Wyrick, 2010). This aberrant state has been termed “persistence.” Importantly, abatement of the stress condition can rescue *Chlamydia* from a persistent state. Limiting the availability of iron to Chlamydiae has long been appreciated as an inducer of persistence, though the exact mechanism that facilitates this development is not understood (Raulston, 1997). Chlamydial persistence may contribute to the development of sub-acute pathologies and *Chlamydia-*associated chronic diseases. Only recently has bona fide *in vivo* evidence provided a clear example of the development of aberrant chlamydial forms in infected women, pigs and mouse models (Pospischil et al., 2009; Phillips Campbell et al., 2012; Lewis et al., 2014). These studies are far from definitive, as they represent small sample sizes and only note the presence of enlarged RB forms among inclusions of mixed populations. Regardless, it remains plausible to hypothesize that the limitation of iron, which induces persistence *in vitro*, may also stimulate aberrant development *in vivo*. Multiple lines of evidence suggest that *Chlamydia* may be exposed to fluctuating iron availability during infection. Specifically for *C. trachomatis* serovars responsible for genital infections (D-K, L1-3), fluctuations in lactoferrin concentrations in the female genital tract in response to estrogen signaling during menstruation could significantly alter iron availability to *Chlamydia* (Cohen et al., 1987; Kelver et al., 1996). Sequestration of iron in the mononuclear phagocyte system (MPS) may represent a host protective mechanism against *Chlamydia* infection by preventing iron trafficking to mucosal epithelia (Nairz et al., 2014; Ganz and Nemeth, 2015; Soares and Weiss, 2015). Iron limitation through the MPS could be especially important for *Chlamydia* species that invade monocytic cells, such as *C. pneumoniae* or *C. trachomatis* lymphogranuloma venereum (LGV) seorvars (L1-3) that presumably invade monocytes to proliferate to the local lymph nodes.

How *Chlamydia* might respond to these nutritive insults remains unclear. In the absence of genetic tools, past studies have relied on the chlamydial genome for clues in this regard. However, the chlamydial genome is relatively small, measuring only 1.04 Mb in *C. trachomatis* (Stephens et al., 1998). Correspondingly, only 894 open reading frames (ORFs) are predicted to be coded on the *C. trachomatis* chromosome. The small genome size of Chlamydiae is accounted for by their long history of co-evolution with specific host cells. This has resulted in severe reductive evolution with respect to the size and functionality of the chlamydial genomic repertoire (Clarke, 2011). *Chlamydia* consequently lack or possess truncated versions of many highly conserved metabolic pathways, such as amino acid and nucleotide biosynthesis. Obligate intracellular parasites are especially susceptible to the accumulation of deleterious mutations, sequence deletions and inversions as they discard functionality from their genome that can be compensated by their hosts (Andersson and Kurland, 1998). This perhaps helps to explain the fact that roughly 32% of the ORFs predicted in the genome of *C. trachomatis* are annotated as hypothetical, with no assigned function based on homology (Stephens et al., 1998). It is therefore no surprise that *Chlamydia* lack many highly conserved components of iron-related systems found in other pathogens.

In this review, we will detail our current understanding of chlamydial iron homeostasis, focusing on both acquisition and regulation, from an historical perspective contextualized with respect to what is understood in other pathogens. This will serve to starkly contrast *Chlamydia* against some of its most similar contemporaries. We also hope to highlight some of the primary difficulties that have stalled progress with respect to our understanding of iron homeostasis in *Chlamydia*. Furthermore, we will discuss some of the recent breakthroughs that we believe will facilitate a more in-depth understanding of iron-related processes in *Chlamydia*.

## Mammalian iron metabolism

Iron metabolism in mammals has been extensively studied and this work has been condensed into comprehensive reviews (for reference see Andrews, 2000; Hentze et al., 2010; Winter et al., 2014). It will suffice here to briefly recount the primary mechanisms by which iron is trafficked to cells, further disseminated throughout the body and how these processes are regulated. Each of these steps represent opportunities for pathogens to siphon iron from their host or for the host to limit iron to pathogens. In humans and all other mammals, ferric iron, obtained through diet, must be reduced before transport across the intestinal epithelium. This process is dependent on a cytochrome b-like ferrireductase (Dcytb) for reduction to the ferrous state (McKie et al., 2001), and on Divalent metal transporter-1 (DMT-1; AKA Nramp2) for transport of the divalent cation into the cytoplasm of the epithelial cell (Fleming et al., 1997, 1998). DMT-1 was shown to transport several different divalent cations, including Mn^2+^ and Zn^2+^, in a proton-coupled symport mechanism (Gunshin et al., 1997). Export across the basolateral membrane of the intestinal epithelium is accomplished via ferroportin-1 (Donovan et al., 2000). Two ferroxidases, ceruloplasmin and hephaestin, are responsible for the re-oxidation of ferrous iron to the ferric form (Osaki et al., 1966; Vulpe et al., 1999; Hellman and Gitlin, 2002). This re-oxidation is important for binding by the serum carrier protein, transferrin (Tf). Tf is a high affinity Fe^3+^ binding protein, with an association constant of approximately 10^36^ for the trivalent metal at a near neutral pH (Ratledge and Dover, 2000). Approximately, 30% of serum transferrin is saturated with iron at any given time, which drives a near immediate sequestration of ferric iron in plasma (Crichton, 2016). Conformational changes occur upon the diferric saturation of transferrin, which, at neutral pH, allows the specific recognition of the diferric-holotransferrin (dfTf) over apo-transferrin at the cell surface by transferrin receptors (TfR) (Cheng et al., 2004). Once bound, these dfTf-TfR complexes localize to clathrin-coated pits and are endocytosed (Harding et al., 1983). In the early endosome, proton pumps cause the decrease of vesicular pH, destabilizing the iron-transferrin complex. While the apo-transferrin-TfR complex is not stable at a neutral pH, at an acidic pH, this complex remains intact and is recycled back to the plasma membrane, where the return to neutral pH causes the dissociation of the apo-Tf, starting the cycle anew (Dautry-varsat et al., 1983). The ferric iron released in the early endosome must be reduced before its transport into the cytosol by DMT-1 (Fleming et al., 1998). This reduction is accomplished by another membrane bound ferrireductase, STEAP3 (Ohgami et al., 2005).

Once in the cytosol, iron is either immediately incorporated into iron utilizing proteins (termed the labile iron pool) or stored by the ferritin complex. Ferritin storage complexes exist as multimers, which are composed of two subunits, designated the heavy (H) and light (L) chains. These subunits surround an iron core that may contain up to 4,500 iron atoms (Harrison and Arosio, 1996; Hentze et al., 2004). The H-subunit possesses ferroxidase activity and can convert the Fe^2+^ molecules into the Fe^3+^ form (Lawson et al., 1989). Little is known about how iron is released from ferritin complexes, although it is assumed that complex degradation functions in this process (Hentze et al., 2004). In erythroid cells, cytosolic iron is used predominantly in the biosynthesis of hemoglobin, which utilizes iron to bind oxygen, and circulate it throughout the body. Indeed, approximately 25 mg of iron per day is devoted to hemoglobin biosynthesis (Hentze et al., 2010). Reduced Fe^2+^ is trafficked to the mitochondrial matrix, the site of heme biosynthesis, where at the last enzymatic step ferrochelatase inserts Fe^2+^ into protoporphyrin IX to form heme (Chung et al., 2012). Importantly, senescent erythroid cells (primarily red blood cells) can be phagocytosed by macrophages which then degrade heme and incorporate the liberated Fe^2+^ into their labile iron pool or direct it to transferrin-mediated trafficking pathways.

Iron homeostasis in mammals is predominantly regulated by one of two mechanisms: The IRP/IRE system or the small peptide hepcidin (Hentze et al., 2010). There are two iron regulatory proteins (IRP1 and IRP2) which function as regulators by binding iron-responsive elements (IRE) in the untranslated regions (UTRs) of mRNA (Rouault, 2006). Notably, IRP1 has a dual function as a cytosolic aconitase when it contains an intact Fe-S cluster, which has led to the “Fe-S switch” model for IRP activation, whereby the regulator is activated in the absence of Fe due to degradation of the Fe-S cluster. Several mRNAs contain IREs, and the regulation of these IREs by IRPs varies. For TfR mRNA, binding of IREs by multiple IRPs stabilizes the mRNA and protects it from endonucleolytic degradation, thus increasing TfR expression (Casey et al., 1989). For ferritin L- and H-chain mRNA, however, IRE binding by a single IRP impedes translation by a steric hindrance mechanism, decreasing ferritin content in the cell (Rouault et al., 1988). IRP1 and IRP2 function redundantly to some extent, as independent knock out models demonstrate significant iron-related phenotypes but double-knockouts are embryonic lethal (Wilkinson and Pantopoulos, 2014). Hepcidin was first thought to be an antimicrobial peptide but was soon found to be critical for proper iron metabolism. Hepcidin was shown to be induced in response to iron overload and hepcidin knock-out mice suffer an iron-overload phenotype, connecting the function of hepcidin to iron homeostasis (Bennoun et al., 2001; Pigeon et al., 2001). It was later shown that hepcidin exerts its effect on iron metabolism mechanistically by binding to and internalizing the iron exporter ferroportin, leading to its degradation (Nemeth et al., 2004). By preventing the transport of iron through the basolateral membrane and thus preventing the dissemination of dietary iron throughout the body, hepcidin has been termed the “master iron regulator.” Interestingly, innate immune signaling induces hepcidin expression and consequently much work has shown that hepcidin is expressed in response to infection by some pathogens, potentially limiting iron from pathogens (Drakesmith and Prentice, 2012). Conversely, in other infection models such as chronic hepatitis C virus infection, hepcidin expression is repressed, and it is thought this may promote iron overload conditions such as hepatic iron accumulation. In total, the circulation of iron through the human body presents many opportunities for pathogens to intervene in their pursuit for iron. Some prominent examples will be discussed below.

## Iron acquisition in intracellular bacterial pathogens

Intracellular pathogens compete for iron sequestered in host proteins, often by intercepting or interrupting mammalian iron trafficking pathways. Iron acquisition follows a general paradigm in intracellular pathogens: Iron, in one form or another, is recognized by the bacteria and translocated across the bacterial membrane(s), generally by a permease system, where it can then be utilized. There are four predominant mechanisms by which this occurs: the biosynthesis of siderophores, transferrin/lactoferrin capture, heme capture, or direct acquisition of ferrous iron. We will briefly recount these mechanisms, but the reader is directed to several detailed reviews on the subject for further reading (Marx, 2002; Andrews et al., 2003; Caza and Kronstad, 2013).

The biosynthesis of small iron-chelating molecules, known as siderophores, represent a primary mechanism of ferric iron acquisition among bacteria. Model siderophores, such as enterobactin, have a notably high affinity for Fe^3+^, (K_a_ = 10^51^), which allows them to directly chelate iron bound in host proteins such as transferrin (Carrano and Raymond, 1979). Siderophores are then transported across the outer membrane by a TonB-dependent receptor protein, bound in the periplasm by a siderophore-binding protein and trafficked through an inner membrane permease system where the siderophore can then be dissociated from Fe^3+^ to facilitate reduction to Fe^2+^. These systems are represented by homologs of *E. coli fepABCDEG* and *fhuABCDEF*, the respective permease systems of enterochelin and ferrichrome. (Fecker and Braun, 1983; Pierce et al., 1983; Ozenberger et al., 1987). The mechanism of TonB-dependent energy transduction from the inner membrane to the outer membrane remains a hotly debated subject of research. What can be concluded from the available data is that the association of TonB with the accessory proteins ExbB and ExbD results in the charging of TonB with potential energy as a function of the proton motive force across the inner membrane (Gresock et al., 2015). Several hypotheses have existed for how the “charged” TonB then delivers energy to the outer membrane receptor proteins which depend on it for the translocation of important nutrients into the periplasm. Recent studies suggest that in contrast to models that posit the shuttling of TonB through the periplasm to the outer membrane, TonB is stationary at the inner membrane and confers energy transduction from this position (Gresock et al., 2011). Most of the iron acquisition systems in Gram-negative bacteria utilize TonB-dependent outer membrane receptors.

In addition to siderophore-mediated acquisition of ferric iron, many Gram-negatives possess mechanisms for the acquisition of ferrous iron, which is presumed to freely flow through the outer membrane, possibly via porins. This strategy is dependent upon ferrireductase activity, which is thought to be either cell-associated or translocated into the extracellular space. Several bacteria have been shown to display ferrireductase activity, but few ferrireductases have been directly identified. Notably, riboflavin has been implicated as a ferrireductase in the Gram-negative pathogens *Campylobacter jejuni* and *Helicobacter pylori* (Worst et al., 1998; Crossley et al., 2007). It was recently shown that *Legionella pneumophila* secretes a pylomelanin pigment that indeed reduces Fe^3+^ to Fe^2+^, and some fungal ferrous iron permease homologs have associated ferrireductases (Kosman, 2003; Chatfield and Cianciotto, 2007). Ferrous iron import systems are generally represented by homologs of *E. coli feoABC* and *Yersinia pestis yfeABCD* (Kammler et al., 1993; Bearden and Perry, 1999). Intriguingly, it has been demonstrated in *Shigella flexneri* that homologs of both ferrisiderophore and ferrous iron uptake systems are induced during host cell invasion and intracellular proliferation (Runyen-Janecky and Payne, 2002; Pieper et al., 2013). This suggests not only that intracellular bacteria experience iron deprivation likely as a protective mechanism of the host, but additionally that direct acquisition of iron ions may represent a “path-of-least-resistance” for intracellular pathogens in their quest for sufficient iron. Moreover, these mechanisms are redundant in *S. flexneri*, indicating that intracellular pathogens have access to both oxidative states of iron in the host cell (Runyen-Janecky et al., 2003). However, because *Shigella* replicates within the host cell cytoplasm (Mellouk and Enninga, 2016), it is not clear if pathogens that reside within intracellular vacuoles could similarly or as effectively utilize these sources of iron.

The serum iron-binding protein transferrin (Tf) is abundant in mammalian blood, and represents an iron resource for extracellular pathogens. Tf is endocytosed by mammalian cells, via holo-Tf recognition by the transferrin receptor (TfR; Harding et al., 1983). Upon endocytosis, TfR-holo-Tf vacuoles are trafficked through the endocytic recycling pathways. This pathway provides an opportunity for intracellular pathogens to obtain holo-Tf and thus iron. *Neisseria* species possess sophisticated mechanisms for the acquisition of transferrin. *Neisseria meningitidis* access host Tf by manipulating Tf-trafficking in the cell, redirecting it to sites of bacterial colonization (Barrile et al., 2015). Transferrin can then be acquired by *Neisseria* via fusion of Tf-containing endosomes with the pathogen-containing vacuole. Two transferrin binding proteins, TbpA and TbpB, are involved in binding and transporting transferrin across the outer membrane in *N. gonorrhoeae* (Cornelissen et al., 1992; Anderson et al., 1994). The two proteins function coordinately, where the outer membrane lipoprotein TbpB acts as a co-receptor to bind transferrin and help direct it to transmembrane TbpA where it can then be transported across the membrane (Ostberg et al., 2013). TbpA is TonB-dependent, the energy from which aids in initiating a conformational change that facilitates the dissociation of apo-transferrin from iron (Noinaj et al., 2012a,b). Free iron can then be transported into the periplasm and bound by the periplasmic ferric binding protein FbpA. The *fbpABC* operon encodes the periplasmic FbpA in conjunction with the inner membrane permease FbpB and the nucleotide-binding FbpC, which facilitate the translocation of Fe^3+^ across the inner membrane (Adhikari et al., 1996).

Most iron in the mammalian body is sequestered in heme molecules. Due to the cytotoxicity of free heme, most heme is bound in hemoproteins such as hemoglobin. Hemoproteins are ubiquitously expressed in mammalian cells, as they play crucial functions in metabolism (e.g., electron transport chain). Thus, hemoproteins and heme represent an ample pool of iron to intracellular pathogens. The acquisition of heme from the host is well-characterized in *Yersinia* species, and is representative of systems found in other pathogens such as *Neisseria* and *Bartonella* (Rohde and Dyer, 2004; Parrow et al., 2009). The *hmuRSTUV* operon encodes the full complement of gene products necessary to receive heme at the outer membrane (HmuR), shuttle it through the periplasm (HmuT), transport it across the inner membrane (HmuUV) and traffic it through the bacterial cytoplasm (HmuS; Thompson et al., 1999). Some pathogens, such as *Brucella abortus, N. meningitidis* and *Bradyrhizobium japonicum* encode heme oxygenases that facilitate the use of heme as an iron source by degrading the tetrapyrrole ring and liberating the coordinated iron (Zhu et al., 2000; Puri and O'Brian, 2006; Ojeda et al., 2012). *Yersinia enterocolitica* encodes an *hmuRSTUV* analog, *hemRSTUV* (Stojiljkovic and Hantke, 1992). Interestingly, *Y. pestis* has a secondary hemophore-dependent heme-protein acquisition system encoded by *hasRADEB* (Carniel et al., 2001). This system functions similarly to both *hmuRSTUV* and *hemRSTUV* with the exception that HasA encodes a secreted heme-binding protein, or hemophore, that can coordinate hemin and shuttle it to the receptor HasR (Lefèvre et al., 2008; Kumar et al., 2013). Intriguingly, essentially none of these receptor mechanisms are identifiable by homology in obligate intracellular pathogens.

Very little is presently understood about iron acquisition and homeostasis in obligate intracellular pathogens. Interestingly, many do not appear to have strict dependencies on iron availability. For example, *Coxiella burnetii* seem to prefer a low-iron environment, potentially as a mechanism to avoid the oxidative stress associated with free iron (Mertens and Samuel, 2012). This is supported by the fact that iron-depleted cell culture systems and animal models actively promote the growth and replication of *C. burnetii* (Briggs et al., 2008). Additionally, only three putative iron-regulated genes appear to be regulated by *C. burnetii* Fur transcriptional repression as determined by a two-plasmid reporter assay (Briggs et al., 2008). Correspondingly, *Rickettsia rickettsii* demonstrate inhibited growth in response to iron-depletion only at very high (500 μM) concentrations of the iron chelator desferrioxamine mesylate (DFO), and only 12 genes appear differentially expressed in response to this stimulus, most of them being hypothetical (Ellison et al., 2009). Moreover, whereas expression of Nramp1 in macrophages affects significant killing of *Salmonella, Coxiella* are resistant to the depletion of metal ions by this transporter (Cockrell et al., 2017). Neither *Coxiella* nor *Rickettsia* have any apparent functional homologs to siderophore biosynthesizing enzymes, but it is interesting to note that *Coxiella* possesses a frameshifted ORF for a siderophore synthase, suggesting that reductive evolution of the genome is actively remodeling the iron requirement of this pathogen (Briggs et al., 2008; Ellison et al., 2008). Contrastingly, *Ehrlichia* species are known to require iron. Both *E. chaffeensis* and *E. sennetsu* fail to proliferate in the presence of only 15 μM DFO and co-localize with TfR to possibly acquire iron (Barnewall et al., 1999). Moreover, supplementation of holo-TfR to *Ehrlichia-*infected THP-1 monocytes treated with the inflammatory cytokine gamma-interferon (IFN-γ) abrogates IFN-γ killing of *E. chaffeensis*, demonstrating the importance of iron availability to *Ehrlichia* pathogenesis (Barnewall and Rikihisa, 1994). Still, despite evidence that iron availability may modulate *Ehrlichia* pathogenesis, the only known iron acquisition protein in *Ehrlichia* is a homolog of FbpA (Doyle et al., 2005). *Chlamydia* species are somewhat distinguished from other obligate intracellular pathogens insofar as the iron-dependency of the organism has been an active subject of research within the field for nearly two decades. Like studies in other bacteria, attempts to dissect iron acquisition in *Chlamydia* began with investigations into chlamydial responses to iron deprivation.

### Chlamydial response to iron starvation

The interest in chlamydial iron biology originated from work done in the laboratory of Jane Raulston, which demonstrated that restricting iron to *C. trachomatis* serovar E-infected epithelial cells by DFO treatment resulted in a significant reduction in infectious progeny and the development of aberrant, morphologically enlarged RBs that exhibited a delayed maturation (Raulston, 1997). Importantly, the addition of holo-Tf to the cell culture medium resulted in the recovery of infectious progeny, indicating that the effect was reversible. These results closely mirrored what had been known for some time with respect to the development of chlamydial persistence in response to IFN-γ treatment (Byrne et al., 1986; Thomas et al., 1993; Beatty et al., 1994). Therefore, based on similarity to IFN-γ-mediated persistence, iron depletion became recognized as an inducer of chlamydial persistence. This discovery prompted research into how exactly *Chlamydia* responds to iron starvation, with the hopes of uncovering molecular mechanisms that function in iron acquisition and homeostasis, and perhaps even persistence. Studies were thus conducted in several other chlamydial species and serovars. Depriving *C. pneumoniae* of iron inhibited growth and resulted in the depletion of infectious progeny and the development of abnormal inclusion and chlamydial morphology (Al-Younes et al., 2001; Freidank et al., 2001). Interestingly, iron chelation seemed to have a more pronounced impact on *C. pneumoniae* than on *C. trachomatis*, as demonstrated by smaller inclusions and markedly fewer recoverable infectious particles following equimolar treatment with DFO. This prompted the investigation of whether iron availability was predictive of *C. pneumoniae-*associated heart disease (Sullivan and Weinberg, 1999). Indeed, samples from patients operated on for stenotic aortic heart valves who also tested positive for *C. pneumoniae* infection showed a greater than 20-fold enrichment for iron, implicating iron availability as a factor in *C. pneumoniae*-associated development of aortic stenosis (Nystrom-Rosander et al., 2003).

Attempts have been made to limit iron to *C. trachomatis* without the use of a chemical chelator, namely by the over-expression of the iron exporter ferroportin (Dill and Raulston, 2007; Paradkar et al., 2008). In Dill and Raulston's report, ponasterone A-inducible ferroportin was over-expressed in HEK293 cells, and no changes in recoverable infectious progeny or morphology were observed in *C. trachomatis* serovar E. In contrast, Paradkar et al. were able to demonstrate that over-expression of ferroportin in HEK293 cells leads to smaller chlamydial inclusions in both *C. psittaci* and *C. trachomatis* infection models. Upon treatment of the infected cells with the major iron regulator hepcidin, inclusion development was rescued, implicating the over-expression of ferroportin as the primary mediator of the phenotype. The authors further demonstrated that this phenotype was reproduced in primary bone marrow macrophages derived from *flatiron* mutant mice, which possess a missense mutation in one allele of ferroportin, resulting in improperly sorted ferrorportin that is retained in intracellular compartments (Zohn et al., 2007). It is not entirely clear why Paradkar et al. obtained such different results from Dill and Raulston's study. However, Paradkar et al. note that in contrast with Dill and Raulston, they included a 24 or 48 h iron-loading step (supplementation of the media with 10 μM ferric citrate) prior to both ferroportin over-expression in HEK293 cells and *C. pisttaci* infection of the *flatiron* mouse-derived macrophages, respectively. Because a non-iron loaded control was not reported in their study, it is not clear the effect that iron-loading may have had on chlamydial growth or host iron homeostasis.

Several studies have investigated changes in gene and protein expression in models of iron limitation for *Chlamydia*. Differential transcript expression analyses revealed significant down-regulation of genes associated with cell wall morphology (*ompA, omcB*) and nucleoid condensation (*hctB*) in *C. pneumoniae*, thought to represent part of the genetic foundation for the hallmark aberrant morphology observed during persistence (Timms et al., 2009). This study also revealed a significant induction of generic stress-response genes in *C. pneumoniae*, such as *htrA* (21.14-fold) and *ahpC* (8.56-fold), encoding a DO serine protease and thioredoxin peroxidase, respectively. In a parallel study on differential protein expression in iron-starved *C. pneumoniae*, marked discrepancies were apparent, such as the 2.3-fold induction of OmpA and unchanged expression of OmcB (Mukhopadhyay et al., 2006). Notable changes were also observed between the transcriptional responses of *C. pneumoniae* and *C. psittaci*, where the transcriptional repressor of developmentally late genes, *euo*, was induced 2.94-fold in *C. pneumoniae*, but repressed nearly 5-fold in *C. psittaci* (Goellner et al., 2006; Timms et al., 2009). It has been proposed that the induction of *euo* late in infection in response to persistence contributes significantly to the inability of *Chlamydia* to continue its normal development (Belland et al., 2003a). It is not entirely clear where the discrepancies between these studies originate. Some amount of disagreement is expected between species, as well as between transcript and protein expression studies. However, these studies only examined the expression of select genes from defined gene ontology groups, as opposed to examining global transcriptomic changes, possibly excluding similarities, and differences. Importantly, the transcriptional studies, predominantly relying on qRT-PCR, normalized their expression data to an internal reference gene, commonly 16S rRNA. It has been demonstrated that this is a less informative methodology for *Chlamydia*, as 16S rRNA expression levels are observed to decrease dramatically in response to global stresses such as those that induce persistence (e.g., IFN-γ; Ouellette et al., 2006). Iron limitation studies in *C. trachomatis* serovar E wherein transcript abundance was normalized to genomic DNA reported no significant difference in the expression of *euo, ompA*, or *omcB* (Dill et al., 2009). However, *ahpC* transcript expression was still found to be moderately up-regulated, and expression was substantially induced at the protein level.

Investigation of the global transcriptomic response of *C. pneumoniae* to an iron starvation model of persistence by microarray analysis revealed a broad down-regulation of transcription machinery, suggesting that a stalled transcriptome may facilitate the development of persistence (Mäurer et al., 2007). This study reported the down-regulation of *omcB* and *hctB*, however *euo* expression was unaffected, contributing further confusion to the body of transcriptomic data. Global proteomic studies in iron-limited *C. trachomatis* models have identified between 19 and 25 proteins induced in response to iron limitation, however it remains unclear whether the majority of these proteins participate directly in iron acquisition or homeostasis (Raulston, 1997; Dill et al., 2009). The identification of chlamydial Hsp60 as iron-regulated did however raise the possibility that iron availability plays a crucial role in sub-acute pathologies of *C. trachomatis*, such as tubal factor infertility, being that patients are often seropositive for chlamydial Hsp60 (Toye et al., 1993; LaRue et al., 2007). Unfortunately, this hypothesis has remained largely unaddressed. An analogous study in an iron-limited *C. pneumoniae* model only identified differential expression among six proteins, most notably the chromosome partitioning protein ParB and the thioredoxin reductase TrxB (Wehrl et al., 2004). Again, the participation of any of the identified iron-responsive proteins in iron homeostasis was unclear.

A potential contributor to the general lack of clarity produced by past studies on chlamydial responses to iron limitation has been the decision to monitor differential gene/protein expression late in infection following prolonged treatments with iron chelators. These extended treatments meant that most comparisons have been made between highly abnormal, persistent chlamydiae and their untreated, replicative and differentiating counterparts. As such, the results from these studies have been harder to interpret given that it is unclear if the phenotypes observed are a true response to iron limitation or if the organisms are simply abnormal. In part this may have been judged to be necessary, as DFO is a relatively inefficient iron chelator. DFO displays a discriminatory binding affinity for the different valence states of iron, having specific preference for ferric over ferrous iron (Keberle, 1964). Moreover, DFO is relatively membrane impermeable, effectively chelating iron only from the extracellular environment and potentially from within lysosomes into which it can be pinocytosed (Lloyd et al., 1991; Richardson et al., 1994; Cable and Lloyd, 1999; Persson et al., 2003; Glickstein et al., 2005). Thus, for an intracellular pathogen such as *Chlamydia*, extensive DFO treatments would be required to elicit sufficient iron starvation. Other iron chelators have been explored in *Chlamydia* infection models, such as desferasirox and deferriprone, which are more membrane permeable than DFO and thus have greater access to intracellular iron pools (Neufeld, 2006; Paradkar et al., 2008). While both chelators display moderate advantages over DFO regarding their ability to reduce host ferritin levels and inhibit growth of chlamydial inclusions, neither chelator was investigated thoroughly enough to suggest that they induced a more robust or rapid response to iron limitation in *Chlamydia*.

The shortcomings of DFO with respect to iron starvation in *Chlamydia* prompted the investigation of a more efficient chelator of iron, leading to the discovery that the membrane permeable 2,2-bipyridyl (Bpdl) was substantially more effective at depriving *Chlamydia* of iron (Thompson and Carabeo, 2011). First, Bpdl is more efficient at promoting an iron-starved “persistent” phenotype as 48 h treatment starting at the time of infection produces enlarged RBs accompanied by the noticeable lack of EBs in the inclusion lumen, whereas an identical treatment of DFO produces inclusions that resemble untreated 24 h post infection (hpi) inclusions. This is reinforced by the observation that Bpdl treatment predictably modulates the expression of persistence markers, *euo* and *omcB*, where *euo* expression remains elevated late in infection and *omcB* expression is suppressed throughout a 48 h time course (Belland et al., 2003b; Ouellette et al., 2006; Timms et al., 2009). DFO, however, fails to significantly alter the expression of either marker for persistence, demonstrating that Bpdl is much more effective at producing a relevant iron starvation phenotype. This agreed with reports from Dill et al. (2009) which showed that DFO treatment did not modulate the transcript abundance of persistence markers. Second, the reduction in recoverable infectious progeny from Bpdl treatment can be reversed not only by the addition of exogenous ferric iron (Fe(III)Cl_3_), but also by the addition of exogenous ferrous iron (Fe(II)SO_4_), demonstrating that Bpdl, unlike DFO, can be saturated, and thus chelate both ferric and ferrous species of iron. Finally, Bpdl induces a much more robust iron starvation transcriptional response than DFO. Treatment with Bpdl significantly induces the expression of two previously identified iron-responsive transcripts, *ahpC* and *devB*, while equimolar DFO treatment does not affect *ahpC* expression and only marginally induces *devB* expression following 30 h of treatment (Dill et al., 2009). In sum, the utility of Bpdl in chlamydial iron starvation studies was demonstrated by its potent induction of persistence, its broader spectrum of iron chelation, and its ability to modulate the expression of known iron-responsive transcripts.

Contrasting research in other bacteria, such as *E. coli* and *Salmonella*, where investigating iron-deprived responses yielded the discovery of many components of iron acquisition and homeostasis, results from the analogous chlamydial studies did not provide the same degree of clarity. This may have been a result of the general disagreement between studies, serovars and species. Alternatively, as the characterization of Bpdl has shown, this may have resulted from non-ideal methodologies. It has therefore required more indirect approaches to begin to understand the molecular basis of iron acquisition in *Chlamydia*.

### Iron acquisition in chlamydia

Analysis of the genome sequence of *C. trachomatis* serovar D reveals that *C. trachomatis* does not possess homologs for heme capture mechanisms, transferrin or lactoferrin receptors, siderophore biosynthesis pathways, or TonB (Stephens et al., 1998). The notable lack of iron-acquisition homologs is a common feature amongst the *C. trachomatis* serovars and species within the *Chlamydiaceae* (Kalman et al., 1999; Read et al., 2000, 2003; Thomson et al., 2008; Grinblat-huse et al., 2011; Mojica et al., 2011; Donati et al., 2014). A protein estimated to be 30 kDa in size with an isoelectric point of 5.5 was originally identified by Jane Raulston in the initial set of 19 iron-regulated proteins differentially expressed in response to extended DFO treatment (Raulston, 1997). However, this was overlooked as a participator in iron acquisition being that another protein which had a molecular weight of roughly 37 kDa and an unusually basic isoelectric point (>9.0) appeared to closely resemble the biochemical properties of the periplasmic Fe^3+^-binding protein FbpA in *Neisseria* (Mietzner et al., 1984). However, a subsequent study addressing the actual identity and activity of this protein was never reported. It wasn't until a subsequent study that aimed to understand which antigenic chlamydial proteins are iron-responsive that the 30 kDa protein was identified as YtgA, the first ORF in the *ytgABCD* operon (Raulston et al., 2007). Based on sequence analysis, YtgA is a 37 kDa substrate-binding periplasmic protein, involved in the trafficking of divalent metals from the outer membrane to an ABC permease system in the inner membrane, closely resembling the Zn^2+^-binding TroA from *Treponema pallidum* (Lee et al., 1999). YtgA migrated with an apparent molecular weight of 30 kDa in both of Raulston's studies, despite its predicted size of 37 kDa. Subsequent investigation regarding the function of YtgA demonstrated that YtgA had a specific affinity for Fe^3+^ *in vitro*, where radiolabeled ^59^Fe^3+^ could not be competed off purified recombinant YtgA when either Mn^2+^ or Zn^2+^ were added to the system (Miller et al., 2009). However, the affinity of YtgA for Fe^2+^ was not reported. Additionally, prolonged exposure to DFO demonstrated that YtgA was more highly expressed 36 hpi and onward compared to untreated controls, but exposure to other insults such as penicillin treatment did not alter YtgA protein expression. It was also observed that YtgA localized predominantly to chlamydial membranes, with a substantial proportion localized within the periplasm, as determined by immunogold labeling. Interestingly, this localization was disrupted in response to DFO treatment, with a pronounced reorganization of YtgA to the cytoplasm, suggesting that iron transport is disrupted during persistence. These localization data confirmed previous experiments that had observed YtgA localized to chlamydial membranes around the periphery of the inclusion by immunofluorescent microscopy (Bannantine and Rockey, 1999). Importantly, in validating Bpdl as a more efficient iron chelator for *Chlamydia*, Thompson and Carabeo observed the induction of *ytgA* transcripts in response to Bpdl treatment (Thompson and Carabeo, 2011). This demonstrated for the first time that *ytgA* was iron-regulated at the transcript level. While these studies suggested a role for YtgA in the acquisition of iron in *C. trachomatis*, it remained unclear from where *Chlamydia* siphoned iron from the host.

Early studies regarding the establishment of the chlamydial inclusion upon invasion monitored the association of classical endosomal markers—such as TfR—with the inclusion as it developed. Endocytosed TfR follows two distinct recycling pathways back to the plasma membrane that are distinguished by their association with particular Rab family GTPases (Mayle et al., 2012). Upon internalization into the early endosome, TfR-containing vesicles are Rab5-positive. These Rab5-positive TfR-containing endosomal vesicles acquire both Rab4 and Rab7 and are promptly recycled to the plasma membrane within 30 min following internalization. However, TfR-containing vesicles can be routed to the sorting endosome, where they acquire Rab11 GTPases. These TfR-containing Rab11-positive vesicles are trafficked back to the plasma membrane with notably slower kinetics, potentially in a Rab4-dependent manner, and represent the slow-recycling pathway of TfR (van der Sluijs et al., 1992; Sönnichsen et al., 2000). Landmark studies in the laboratories of Ted Hackstadt, Joanne Engle and Thomas Meyer demonstrated that TfR-containing vesicles localize around the chlamydial inclusion in both *C. trachomatis* and *C. pneumoniae-*infected epithelial cells (Scidmore et al., 1996; van Ooij et al., 1997; Al-younes et al., 1999). Interestingly, these studies did not conclusively demonstrate that *Chlamydia* infection modulates the recycling of TfR, suggesting the inclusion association with endocytic vesicles may be fluid and dynamic. Moreover, Scidmore et al. reported that Tf localized to the inclusion periphery but never within the inclusion lumen, eliminating the possibility that *Chlamydia* directly acquire Tf. However, the relation of TfR localization around the chlamydial inclusion to the success of the pathogen was not addressed in these studies.

A later study identified a small molecule that specifically inhibits the slow-recycling pathway of transferrin by preventing Tf-containing Rab11-positive vesicular fusion with Rab4-positive vesicles on their way back to the plasma membrane (Ouellette and Carabeo, 2010). This molecule was identified initially because of its potent anti-chlamydial activity. The inhibition of Rab11-Rab4 hybrid vesicle formation effectively stalled Tf-recycling at the chlamydial inclusion, leading to the accumulation of Tf-containing vesicles around the inclusion membrane. Chlamydial killing was likely caused by exposure to inhibitory amounts of free iron, as removing the transferrin-containing fraction from the serum alleviated inhibition of chlamydial growth. While this study did not directly observe iron transport into the chlamydial inclusion, it determined that normal development of *Chlamydia* is strictly dependent upon the uninhibited recycling of Tf through the slow-recycling endosomal pathway. This finding coincides with the result of an earlier study that observed knocking down expression of Rab11, and thus disrupting Tf recycling, blocks the development of infectious progeny in *C. trachomatis* (Lipinski et al., 2009). Therefore, normal chlamydial development is dependent upon association with Rab11-positive endosomes, which are critical for intracellular trafficking of Tf. *Mycobacterium avium*-containing vacuoles also interact with Rab11-positive organelles, including the endocytic recycling compartments (ERC), but the biological significance of this interaction is unknown (Halaas et al., 2010). *M. tuberculosis* binds holo-transferrin on its surface via a number of proteins, including glyceraldehyde-3-phosphate dehydrogenase (GAPDH). After binding, transferrin is internalized into the bacterial cell (Boradia et al., 2014). The interaction of the Mtb-containing vacuole with ERC-derived Rab11-positive vesicles would be consistent with this transferrin capture strategy. Indeed, such an association was reported during infection of macrophages (Tailleux et al., 2003).

The requirement of Rab11-positive vesicles may help explain why Dill and Raulston's attempt to phenocopy iron starvation in *Chlamydia* by ferroportin over-expression failed (Dill and Raulston, 2007). While ferroportin exports cytosolic iron, *Chlamydia* utilizes intravesicular iron prior to their transport to the cytosol. Chlamydial iron acquisition is a process independent, at least in part, of the dissemination of Fe^2+^ from the endosome into the cytosol (e.g., by the activity of STEAP3 ferrireductase and DMT1 transporter). If so, it would not be expected that increased iron export would impact *Chlamydia* in the absence of unaltered endocytic TfR internalization. These data help support the notion that the chlamydial inclusion functions as a pathogen-specified parasitic organelle, siphoning resources in homeostasis with the subcellular trafficking pathways of the host (Moore and Ouellette, 2014). It is worth mentioning here that it has been reported that *Chlamydia* can propagate in serum-free media, i.e., media lacking transferrin (Maass et al., 1993; Ouellette and Carabeo, 2010). However, the biological significance of this is unclear given that iron is predominantly sequestered in host proteins such as transferrin and ferritin *in vivo*, and eukaryotic host cells uptake iron not in the free elemental form, but bound in macromolecular complexes. Therefore, the concentration of free iron under normal conditions is already likely quite low. This may however indicate that other mechanisms are at work beyond the acquisition of iron via vesicular trafficking of transferrin.

**Figure 1:**
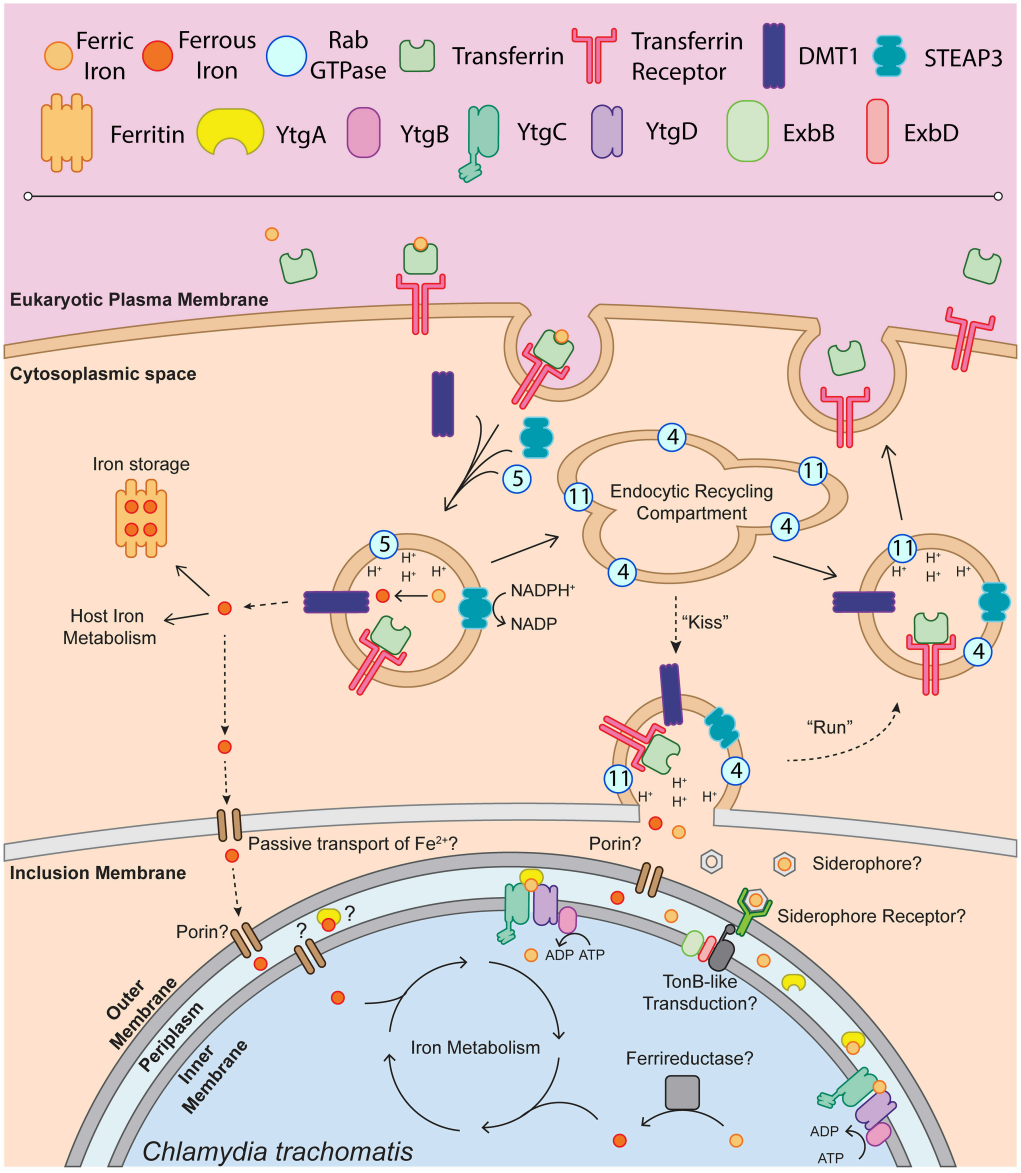
Schematic of chlamydial iron acquisition. Solid black arrows denote iron trafficking pathways of the host whereas dashed-line arrows indicate possible mechanisms of iron acquisition by *Chlamydia*. The depiction of endosome-associated Rab-family GTPases is not intended to be exhaustive and is based on the findings of Ouellette and Carabeo (2010). Ferric iron (Fe^3+^) is bound by apo-transferrin (Tf) and endocytosed upon recognition by the transferrin receptor (TfR). The holo-Tf-TfR complex proceeds into the early endosome, upon which TfR-containing endosomes acquire DMT1, STEAP3, and Rab5. Acidification of the early endosome potentiates the dissociation of Fe^3+^ from Tf, which can then be reduced to ferrous iron (Fe^2+^) by STEAP3. Fe^2+^, exported from the endosome by DMT1 into the cytoplasm, is shunted to ferritin for iron storage or incorporated into host iron metabolic processes such as the electron transport chain or TCA cycle. It is possible that labile Fe^2+^ is passively transported through the inclusion membrane and the chlamydial outer membrane to be sequestered in the periplasm and trafficked into the bacterial cell. A fraction of the TfR-containing endosomes will acquire Rab11 and Rab4, proceeding through the slow recycling endosomal pathway to be trafficked back to the eukaryotic plasma membrane. In an *Chlamydia*-infected cell, slow recycling Tf-containing endosomes associate with the chlamydial inclusion, where a “kiss & run” mechanism may deliver free ferric or ferrous iron into the inclusion lumen. Novel siderophores, siderophore receptors or porins may function to traffic iron to the periplasm. This outer membrane transport may be dependent upon TonB-like energy transduction, given that *Chlamydia* possess ExbB and ExbD homologs. Once inside the periplasm, Fe^3+^ can be bound by YtgA and shuttled to the membrane permease likely formed by YtgC and YtgD. ATP hydrolysis mediated by YtgB likely provides the energy to import iron into the chlamydial cytosol. The reduction of Fe^3+^ to biologically useful Fe^2+^ may be performed by an unidentified ferrireductase. Upon acquisition of Fe^2+^, *Chlamydia* can then incorporate iron into the electron transport chain via porphyrin biosynthesis, divert iron to Fe-S cluster biosynthesis or utilize iron in other metabolic processes.

Taken together, the present data allow the construction of a model by which *Chlamydia* may acquire iron (Figure 1). This model proposes that upon receptor-mediated endocytosis of Tf bound to TfR, a small portion of Tf is trafficked in Rab11-positive vesicles to the periphery of the chlamydial inclusion, possibly where a “kiss-and-run”-type mechanism may facilitate the entry of free iron into the inclusion lumen. In the absence of a ferrireductase homolog in the chlamydial genome, it is possible that both Fe^2+^ (reduced by STEAP3) and Fe^3+^ diffuse into the inclusion lumen upon vesicular fusion with TfR-containing Rab11-positive endosomes. However, it has been suggested that riboflavin may act to reduce Fe^3+^, as riboflavin biosynthetic genes (*ribBA, ribC*, and *ribH*) have been reported to be induced by 1 h post-infection (Humphrys et al., 2013). However, no additional experimental data exists to support this hypothesis. Iron transport across the outer-membrane could be mediated by an as yet unidentified siderophore/receptor mechanism that funnels Fe^3+^ to YtgA. The existence of unidentified siderophores is in part supported by the observation that neutrophil gelatinase-associated lipocalin (Lcn-2) is involved in host-mediated suppression of chlamydial growth (Bellmann-Weiler et al., 2013), possibly via sequestering iron-loaded bacterial siderophores, although siderophore candidates would have to be novel or non-traditional (Flo et al., 2004; Berger et al., 2006; Nairz et al., 2009). Siderophore uptake may be dependent upon a system of energy transduction, as *Chlamydia* possess both ExbB and ExbD, the canonical TonB accessory proteins. However, the evidence for such a system is highly circumstantial at present, as no homolog for siderophore biosynthesizing enzymes or TonB have been identified (Stephens et al., 1998). Ferrous iron may passively diffuse through outer membrane porins into the periplasm. YtgA can then traffic Fe^3+^, and possibly Fe^2+^, to the inner membrane heterodimeric permease likely formed by YtgC and YtgD. The ATP-binding YtgB provides the energy to transport iron across the inner membrane by ATP hydrolysis to ADP. If transported, Fe^3+^ must be reduced by an unidentified chlamydial cell-associated ferrireductase analog to become biologically utilizable. An alternate scenario may provide *Chlamydia* with ferrous iron, exported by DMT1 from the TfR-containing endosomes to the host cell cytosol. This cytosolic pool of unliganded iron could be passively transported across the inclusion membrane and chlamydial outer membrane to be sequestered by YtgA or some other divalent metal binding protein. However, as the ferroportin over-expression system demonstrated, this alternate pathway may not provide the bulk of iron to *Chlamydia*. In addition, cytosolic Fe^2+^ would likely be captured by host ferritins, intensifying competition for this iron pool. Further, *Chlamydia* may access the intracellular labile iron pool. For example, it is understood that the chlamydial inclusion associates with lysosomes to acquire nutrients such as oligopeptides, and *Chlamydia* may further interact with mitochondria and autophagosomes for acquisition of nutrients (Matsumoto et al., 1991; Al-Younes et al., 2004; Derré et al., 2007; Ouellette et al., 2011). However, without properly identifying the subcellular location of the labile iron pool accessed by *Chlamydia*, and in the absence of straight-forward tools to interrogate the interaction between *Chlamydia* and the labile iron pool, questions regarding chlamydial access of the labile iron pool remain difficult to address. The available data thus provides a testable framework within which to continue investigations into chlamydial iron acquisition and begin to delineate which model of acquisition predominates in *Chlamydia*.

## Bacterial iron-dependent transcriptional regulation

That iron availability represents a critical developmental parameter for most organisms implies that the pathways that utilize it must be under tight regulation. Iron-dependent regulation, therefore, has been widely researched in many organisms, including pathogenic bacteria. Following the isolation of mutant strains of *E. coli* and *S. typhimurium* that constitutively expressed iron acquisition machinery, it has been appreciated that bacteria globally regulate iron acquisition in response to iron availability (Ernst et al., 1978; Hantke, 1981). The strains isolated in these studies were classified as *fur* mutants, reflecting the observation that ferric iron uptake was dysregulated. It was later concluded, based upon complementation studies, that the *fur* locus operated as a negative regulator of iron acquisition (Hantke, 1982). The specific repressor activity of Fur is dependent upon Fe^2+^ availability and even early studies were elucidating the role of Fur in *E. coli* virulence (Bagg and Neilands, 1987; Calderwood and Mekalanos, 1987). Fur is now recognized as an iron-dependent master regulator in *E. coli* and many other pathogens, the diverse roles of which have been reviewed extensively (Hantke, 2001; Lee and Helmann, 2007; Carpenter et al., 2009). The Fur family of repressors includes many members for which the repressor activity is dependent upon another divalent cation, such as the Zn^2+^-dependent Zur in *E. coli* and *B. subtilis* or the Ni^2+^-dependent Nur in *S. coelicolor* (Gaballa and Helmann, 1998; Outten et al., 2001; Ahn et al., 2006). Due to the instability of Fe^2+^ in aerobic environments, many other divalent cations, including Mn^2+^, Co^2+^ and Cu^2+^ have been used in biochemical studies of Fur (de Lorenzo et al., 1987). Fur is known to bind to a consensus “Fur-box” sequence of 19 nucleotide bases forming a palindrome (de Lorenzo et al., 1987). Fur has been shown to act both as a repressor and an activator, in both Fe^2+^-dependent and independent ways. However, these activities are not strictly or uniformly dependent on the consensus Fur box sequence. The general mechanism for Fur repression is based on competition with the RNA Polymerase holoenzyme (RNAP), where bound Fur prevents RNAP from associating with a given promoter region (Escolar et al., 1997, 1998; Pohl et al., 2003). Alternatively, when activating transcription, Fur can presumably facilitate the association of RNAP at a target promoter by binding upstream of the promoter element, thus leaving the association of RNAP uninhibited (Alamuri et al., 2006; Gancz et al., 2006). It has also been established that Fur autoregulates its own expression, directly by binding its own promoter region and indirectly by regulating the expression of the small non-coding RNA RyhB, which prevents Fur translation (Vecerek et al., 2007). Notably, Fur negatively regulates RyhB expression and thus indirectly regulates RyhB targets, which are predominantly positively regulated, including components of the TCA cycle and bacterial ferritin proteins (Gottesman and Masse, 2002). The biological insight gained from studies on Fur inspired investigations into other metal dependent regulators in bacteria, of which the iron-dependent DtxR has emerged as the opposing family-defining member.

The existence of the DtxR regulator was postulated as far back as 1974 as a possible mechanism for controlling the iron-dependent expression of the diphtheria toxin in *Corynebacterium diphtheriae* (Murphy et al., 1974). Isolation of the *dtxR* locus and characterization of its activity against the diphtheria toxin (*tox*) promoter demonstrated that DtxR functions as a negative regulator of *tox* expression in an iron-dependent manner, while being unable to functionally complement repression of Fur-regulated promoters (Boyd et al., 1990). Accordingly, Fur is incapable of repressing *tox* promoter-driven reporter expression, suggesting that Fur and DtxR are not complementary repressors. This coincides with the low nucleotide and amino acid sequence homology observed between the two repressors. Purified DtxR binds the *tox* promoter region in the presence of either Fe^2+^, Ni^2+^, Co^2+^, or Mn^2+^ and is dependent upon a nine base-pair interrupted palindrome (Schmitt et al., 1992; Tao et al., 1992). DtxR additionally regulates siderophore biosynthesis in *C. diphtheriae* along with several other iron-regulated promoter regions (Schmitt and Holmes, 1991, 1994; Lee et al., 1997; Kunkle and Schmitt, 2005). Importantly, a 19 bp consensus sequence for DtxR regulation has been defined, and exhibits limited sequence homology to the Fur consensus sequence (Schmitt and Holmes, 1994). Functional studies have demonstrated that DtxR has two important metal binding sites, with metal site 2 being essential for activation (Spiering et al., 2003). Interestingly, metal binding appears to facilitate homo-dimer stability, demonstrating how DtxR may function as an iron-sensor protein via an innate dependence on iron for dimerization and thus repressor activity. DtxR dimers aggregate around DNA in groups of two and, similarly to Fur, prevent RNAP association at the promoter region. *M. tuberculosis* encodes an iron-dependent DtxR-homolog annotated IdeR that appears to function analogously to DtxR in coordinating iron homeostasis via transcriptional repression, but appears to additionally act as a transcriptional activator (Gold et al., 2001). TroR in *T. pallidum* is a Mn^2+^-dependent transcriptional repressor, which autoregulates its own operon, but little else is understood about its broad regulatory function (Posey et al., 1999). Similarly, both *C. diphtheriae* and *B. subtilis* encode Mn^2+^-dependent MntR, which regulates manganese homeostasis by transcriptional repression under high manganese conditions (Que and Helmann, 2000; Schmitt, 2002). Interestingly, *B. subtilis* MntR also activates transcription of the *mntABCD* operon under low manganese conditions. Importantly, Fur- and DtxR-like family members can function cooperatively, such as in *E. coli* where both Fur and MntR coordinately regulate the expression of manganese uptake (Patzer and Hantke, 2001).

Unlike Fur and DtxR, the activity of which is modulated by coordination of Fe^2+^, many α-proteobacteria such as *B. japonicum* possess iron response regulators (Irr) that are instead regulated by the availability of heme (Hamza et al., 1998). The association of ferrocheletase with Irr inactivates the protein after which heme binding facilitates the degradation of Irr (Qi et al., 1999; Qi and O'Brian, 2002). Irr is expressed alongside a Fur homolog in *B. japonicum*, which represses *irr* expression in a Mn^2+^-dependent manner, while Irr itself acts as an iron-dependent antirepressor of *irr* expression (Hamza et al., 2000; Hohle and O'Brian, 2010). Irr has been shown to function both as an iron-dependent transcriptional repressor and activator in *B. japonicum* (Sangwan et al., 2008; Small et al., 2009). Irr regulation is generally dependent on a cis-acting iron control element (ICE) in the promoter region of target genes, consisting of an AT-rich 21 bp incomplete inverted repeat (Nienaber et al., 2001; Rudolph et al., 2006). The putative Irr-dependent regulon includes many genes associated with iron homeostasis, including heme-uptake (*hmuR, hmuT*, etc.) and iron receptor proteins (*fcuA*, blr3904; Rudolph et al., 2006). In sum, bacterial metallo-regulation is remarkably diverse, functioning in multiple capacities to control a wide array of pathways. Correspondingly, it is important to thoroughly interrogate putative metallo-regulators with respect to these various functions, including the examination of multiple possible metal cofactors, regulatory activities (i.e., negative vs. positive regulation, direct vs. indirect regulation) and prospective regulons. With respect to chlamydial metallo-regulation, we are only just beginning to tackle each of these criteria.

### Chlamydial iron-dependent regulation

The early studies of chlamydial responses to iron deprivation invigorated interest in identifying the underlying modes of gene regulation. However, sequence homology did not indicate the existence of an obvious homolog to known iron-dependent regulators such as Fur, DtxR or Irr in the chlamydial genome. In fact, to date only eight transcriptional regulators have been characterized in *Chlamydia*, many of which were not immediately identified by sequence homology (Wyllie and Raulston, 2001; Wilson and Tan, 2002; Carlson et al., 2006; Koo et al., 2006; Schaumburg and Tan, 2006; Case et al., 2011; Rosario and Tan, 2012; Thompson et al., 2012). Moreover, no iron-dependent post-transcriptional mechanisms, such as RyhB homologs, have been identified. Technologies to mutagenize or transform *Chlamydia* to screen for iron-related phenotypes have only recently been developed and as such were not viable research strategies to address iron regulation initially. Despite these hurdles, Wyllie and Raulston endeavored to identify candidate iron-dependent regulators by more rigorously surveying the available sequence data. CLUSTAL sequence alignment revealed five ORFs that demonstrated limited sequence homology to Fur in other Gram-negative bacteria (Wyllie and Raulston, 2001). Among these five ORFs, CT296 emerged as a strong candidate Fur homolog following several lines of evidence: CT296 was specifically recognized by *Pseudomonas aeruginosa* Fur-specific polyclonal antiserum, ectopic expression of recombinant CT296 complemented a Fur-repression phenotype in *E. coli*, and CT296 bound a consensus Fur-box sequence in an electrophoretic mobility shift assay (EMSA). CT296 co-migrated with DNA in the presence of both Zn^2+^ and Mn^2+^; however, Fe^2+^ was not assayed in this study due to precipitation of the protein upon incubation with the biometal. Together, these data prompted the renaming of CT296 to divalent cation-dependent regulator (DcrA), and represented the first characterized transcriptional repressor in *Chlamydia*. These studies were then extended when a subsequent investigation utilized a Fur titration assay (FURTA) screen to identify regions of the chlamydial genome recognized by *E. coli* Fur (Rau et al., 2005). Selected candidate Fur-binding sequences were then screened for DcrA interaction by EMSA, identifying *CT733, mreB, glgP*, and *tolR* as possible DcrA regulatory targets. This work held the promise of beginning to define the scope of metallo-regulation in *Chlamydia*. However, later structural modeling, both by *ab initio* computational modeling and X-Ray crystallography, revealed that CT296 displayed no structural similarity to Fur and moreover contained no conserved DNA-binding motifs (Kemege et al., 2011). Furthermore, this study could not reproduce both the EMSA and the Fur-complementation assays previously reported by Wyllie and Raulston. Unfortunately, the function of CT296 has not been explored in further depth.

Perhaps unsurprisingly, the identification of a subsequent metallo-regulator in *Chlamydia* again required the implementation of a more specific and tailored examination of the available sequence data. By analyzing 240 predicted ORFs from the *C. trachomatis* serovar D genome using an *in silico* functional assignment algorithm, CT069 was identified as a candidate transcriptional regulator containing a C-terminal DtxR-like HTH domain (Akers et al., 2011). This was a surprising discovery given that *CT069* encodes YtgC, annotated as an integral membrane permease that functions in concert with the *ytgABCD* operon. More careful inspection of the YtgC sequence revealed that this assignment was based on sequence homology of the N-terminus of YtgC to TroC in *T. pallidum*, but interestingly, the C-terminus possesses high identity to TroR, the Mn^2+^-dependent transcriptional repressor of the *troABCDR* operon (Posey et al., 1999). Akers et al. characterized the function of this C-terminal domain, YtgR, by demonstrating that YtgR could bind and repress the *ytg* promoter region in a Zn^2+^-dependent fashion *in vitro* and in a heterologous *E. coli* system. Curiously, this study reported on the activity of YtgR in the presence of Zn^2+^ and Mn^2+^, but not Fe^2+^. Given that the expression of YtgA was previously demonstrated to be iron-responsive and functioned to specifically bind Fe^3+^ over *both* Mn^2+^ and Zn^2+^, there existed strong evidence that the *ytg* system functioned in iron transport (Miller et al., 2009). The Zn^2+^-dependent function of YtgR did not appear to correspond with what was known about the *ytgABCD* operon and the authors did not provide an explanation as to how zinc availability may influence iron acquisition. It is plausible that the *ytgABCD* permease functions as a broad-spectrum divalent metal importer, and thus may be regulated by Zn^2+^ and import various divalent cations, but this was not addressed in this study. Regardless, the decision not to address the potential iron-dependency of YtgR left questions unanswered, especially considering the recognized variety of metal corepressors documented in Fur and DtxR activity. In addition, Akers et al. did not directly address how this YtgC-YtgR fusion could function as a transcriptional repressor if YtgC was indeed sequestered in the chlamydial inner membrane.

Independently, it was observed that the expression of *ytgA* at the transcript level was significantly induced in response to iron chelation by 2,2-bipyridyl, which was discussed in detail above (Thompson and Carabeo, 2011). This observation added more evidence to support the hypothesis that A.) the *ytgABCD* operon functions in iron transport and B.) that the *ytgABCD* operon was controlled by an iron-dependent system of regulation. Therefore, Thompson et al. conducted independent *in silico* analyses and confirmed the presence of the C-terminal DtxR-like domain within the YtgC coding sequence (Thompson et al., 2012). Importantly, critical DtxR metal-coordinating residues were highly conserved in the YtgC repressor domain (Pohl et al., 1999). Accordingly, the authors re-evaluated YtgR repressor function, to address both the ability of the repressor to utilize Fe^2+^ as a corepressor and act as a transcriptional repressor when physically fused with an integral membrane domain. Thompson *et al*. demonstrated that YtgR specifically bound the *ytg* promoter region in a Fe^2+^-dependent manner by *in vitro* repressor-DNA interaction assays. Importantly, YtgR was screened for DNA-binding activity against an assortment of divalent cations, including zinc, manganese, copper and cobalt, and observed no YtgR binding to the promoter with any of these possible alternative corepressors. This was in clear disagreement with the Zn^2+^-dependent activation of YtgR reported by Akers et al. It is possible that Zn^2+^ confers activation of YtgR at a higher concentration, and thus the DNA-binding assay employed by Thompson et al. simply failed to detect this activation. This would partly agree with the observation that equimolar concentrations of Zn^2+^ only partially activate DtxR DNA-binding in comparison with Fe^2+^ (Tao and Murphy, 1992). On that point, it is worth noting that Akers et al. relied on an *E. coli* Zn^2+^-efflux mutant (*zntA zitB*) to demonstrate Zn^2+^-dependent YtgR repression *in vivo*, concentrating Zn^2+^ in their heterologous experimental system. Given that some metallo-regulators, such as *B. japonicum* Mur and Fur, alter their metal affinity and thus repressor activity in different cellular environments (Hohle and O'Brian, 2016), it is unclear how artificially increased Zn^2+^ concentrations may affect YtgR metal specificity and activity. Ultimately, the new data reported by Thompson et al. suggested a strong preference for Fe^2+^ in YtgR activation.

Thompson et al. additionally observed that YtgR undergoes cleavage from YtgC during infection, as CT069 (YtgC) antisera detects two bands which migrate at approximately 49 and 28 kDa, likely representing the uncleaved YtgC and the YtgR cleavage product, respectively. Intriguingly, in purified EB preparations, only the 28 kDa species is detectable, suggesting that the EB is primed for iron-dependent regulation upon invasion. In agreement with the results obtained by Akers et al. the authors demonstrated that ectopically expressed recombinant YtgR can repress transcription from the *ytg* promoter in a heterologous *E. coli* system. Importantly, however, it was also demonstrated that ectopically expressed recombinant full-length YtgC displayed the same phenotype, suggesting that functional YtgR was cleaved from YtgC in this heterologous system. Indeed, this hypothesis was supported by immunoblot analysis of ectopically expressed recombinant YtgC in *E. coli*, which revealed several C-terminal cleavage products, a likely consequence of non-specific cleavage. Despite non-specific cleavage events, the *E. coli* reporter assay suggests that at least one of the cleaved products acted to repress transcription from the *ytg* promoter. It is possible that this data indicates that full-length YtgC can also function as a transcriptional repressor, perhaps similarly to membrane-anchored LacI (Görke et al., 2005). However, given that the C-terminal domain is liberated endogenously, cleavage likely represents a critical step in the processing of the repressor domain. Intriguingly, the authors identified the presence of orthologous permease-repressor fusion proteins in 11 bacterial phyla, suggesting that this mechanism of transcriptional regulation may exist throughout the bacterial kingdom.

**Figure 2:**
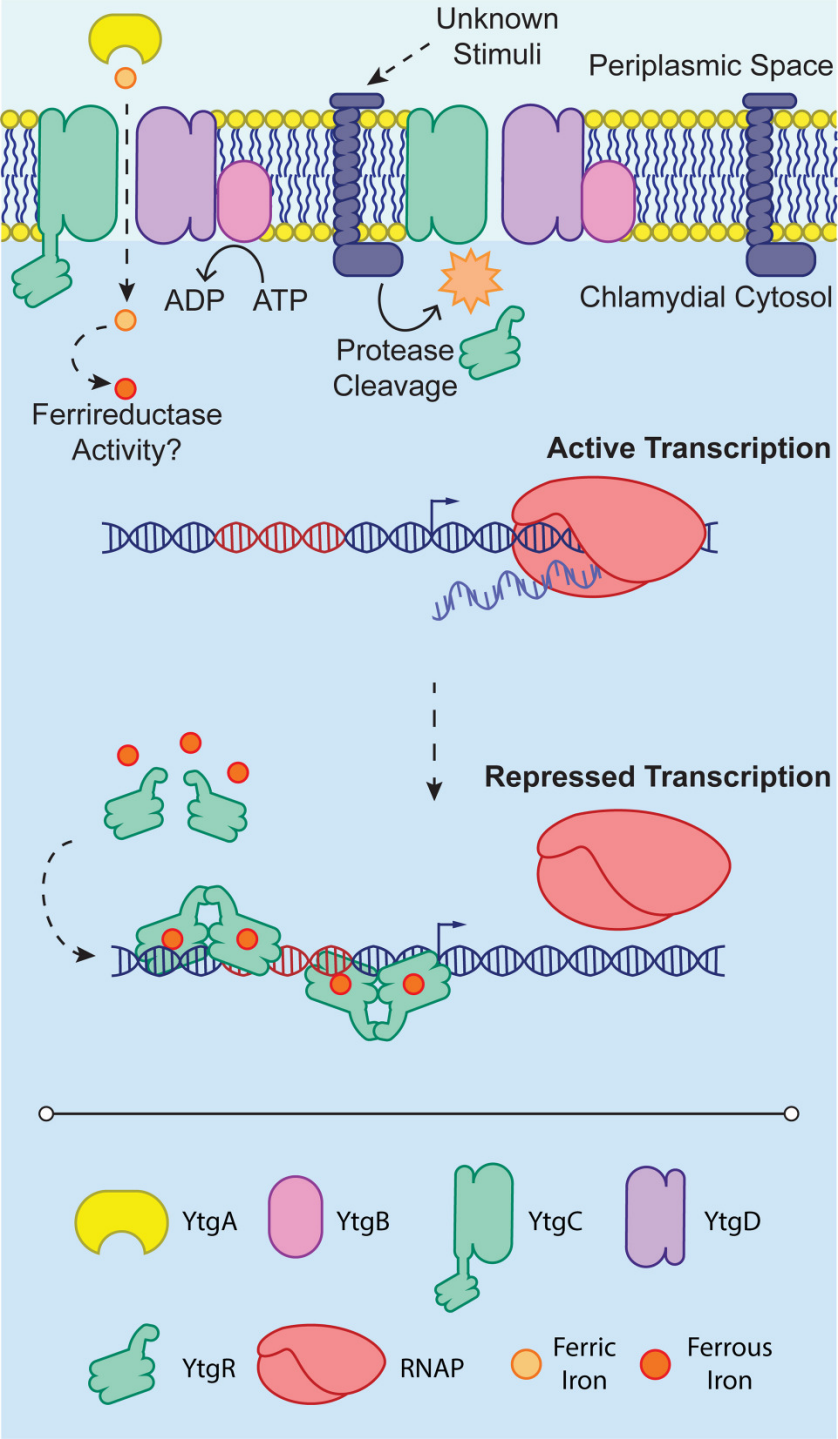
YtgR-mediated transcriptional regulation. To enable regulation, YtgR must be cleaved from the integral membrane YtgC domain. This cleavage is poorly understood and is depicted here as an integral membrane protease only for convenience. The stimuli that induce cleavage of YtgC in *Chlamydia* are not known. Under low iron conditions, expression of the *ytgABCD* operon is uninhibited, and the RNA polymerase holoenzyme (RNAP) can associate with the promoter region and initiate transcription. In contrast, when iron concentrations are sufficiently high, presumably following high expression of the *ytgABCD* operon and thus increased iron uptake, Fe^2+^ can activate YtgR-dependent transcriptional repression. This may occur by the association of pairs of YtgR homodimers binding to the operator sequence (depicted in red) as has been observed for DtxR. Transcriptional repression is thus accomplished by occlusion of RNAP from the promoter region of the operon.

Thus, the present model for iron-dependent regulation in *Chlamydia* involves first the import of iron through the Ytg permease system, which allows, upon the accumulation of sufficient iron, cleaved YtgR to coordinate Fe^2+^ and subsequently autoregulate the expression of the iron uptake machinery likely by out-competing RNAP for DNA binding (Figure 2). While cleavage of YtgR from YtgC was observed, a protease responsible for this cleavage has not yet been identified. However, the observation that YtgC is also cleaved heterologously in *E. coli* may indicate that the culprit protease is represented by a chlamydial homolog of a known *E. coli* protease. The stimulus that induces YtgC cleavage and thus liberation of YtgR has not been described. It is worth noting, however, that regardless of the exact stimulus, the requirement of cleavage imposes an additional level of regulation on chlamydial iron homeostasis at the level of the protease. The protease in turn may be regulated, further extending the tight control *Chlamydia* exerts on iron acquisition. At present, it is not clear how YtgR binding of DNA accomplishes transcriptional repression. As a DtxR homolog, YtgR may similarly complex around operator sites as a couplet of homodimers (Pohl et al., 1999). However, this model will require direct investigation of YtgR binding ratios and structural analyses to be verified. The identification of YtgR as an iron-dependent transcriptional repressor should potentiate subsequent studies of the iron regulon in *Chlamydia*, allowing researchers to address both functional and categorical aspects of iron-dependent regulation in a more systematic manner.

## Concluding remarks

For 20 years it has been understood that iron represents a critical nutrient for the proper development of *Chlamydia*. Only in the latter half of this period have we begun to cement our understanding of chlamydial iron homeostasis in terms of molecular mechanisms. Thus, we can now place the *ytgABCD* operon squarely at the center of our knowledge regarding iron acquisition in *Chlamydia*. This agrees with the iron-dependent autoregulatory function of the cleavage product of YtgC, the YtgR repressor domain. Recent data also strongly suggests that iron is trafficked to the chlamydial inclusion via the slow recycling pathway of transferrin. Moreover, we now have more adequate tools with which to address important questions surrounding chlamydial iron biology. The iron chelator 2,2-bipyridyl should facilitate more robust studies on the responses of *Chlamydia* to iron starvation. The advantages of bipyridyl over previous methods may provide the opportunity to begin to truly understand the primary response of the organism to iron deprivation, as compared to the response of fully persistent aberrant chlamydial forms. In addition, as the golden age of chlamydial genetics has now dawned upon the field, we can ask more prying questions into the molecular basis underlying chlamydial iron responses by manipulating chlamydial, as well as host proteins, and understand in greater detail the iron-dependent regulon of *Chlamydia* and the ways in which *Chlamydia* acquires and traffics iron away from their host. Fully elucidating the regulatory function of YtgR and the mechanism of the *ytg* iron acquisition system will likely require the introduction of subtle mutations, such as base substitutions, given the potential indispensability of these systems for proper chlamydial development. Technologies such as CRISPR/Cas9 will be invaluable in these studies. *Chlamydia* appears to defy stereotypes at every turn, and we can no longer make assumptions on mechanisms or simply rely on referencing systems present in other organisms if we are to understand the basic biology of this pathogen.

## Author contributions

All authors listed have made a substantial, direct and intellectual contribution to the work, and approved it for publication.

### Conflict of interest statement

The authors declare that the research was conducted in the absence of any commercial or financial relationships that could be construed as a potential conflict of interest.

## References

[B1] AbdelRahman Y. M. Belland R. J. (2005). The chlamydial developmental cycle. FEMS Microbiol. Rev. 29, 949–959. 10.1016/j.femsre.2005.03.002 16043254

[B2] Adhikari P. Berish S. A. Nowalk A. J. Veraldi K. L. Morse S. A. Mietzner T. A. (1996). The fbpABC locus of *Neisseria gonorrhoeae* functions in the periplasm-to-cytosol transport of iron. J. Bacteriol. 178, 2145–2149. 10.1128/jb.178.7.2145-2149.1996 8606197 PMC177918

[B3] Ahn B. Cha J. Lee E. Han A. Thompson C. J. Roe J. (2006). Nur, a nickel-responsive regulator of the Fur family, regulates superoxide dismutases and nickel transport in *Streptomyces coelicolor*. Mol. Microbiol. 59, 1848–1858. 10.1111/j.1365-2958.2006.05065.x 16553888

[B4] Akers J. C. HoDac H. Lathrop R. H. Tan M. (2011). Identification and functional analysis of CT069 as a novel transcriptional regulator in Chlamydia. J. Bacteriol. 193, 6123–6131. 10.1128/JB.05976-11 21908669 PMC3209234

[B5] Alamuri P. Mehta N. Burk A. Maier R. J. (2006). Regulation of the *Helicobacter pylori* Fe-S cluster synthesis protein NifS by iron, oxidative stress conditions, and Fur. J. Bacteriol. 188, 5325–5330. 10.1128/JB.00104-06 16816209 PMC1539971

[B6] Al-Younes H. M. Brinkmann V. Meyer T. F. (2004). Interaction of *Chlamydia trachomatis* serovar L2 with the host autophagic pathway. Infect. Immun. 72, 4751–4762. 10.1128/IAI.72.8.4751-4762.2004 15271937 PMC470602

[B7] Al-younes H. M. Rudel T. Meyer T. F. (1999). Characterization and intracellular trafficking pattern of vacuoles containing *Chlamydia pneumoniae* in human epithelial cells. Cell. Microbiol. 1, 237–247. 10.1046/j.1462-5822.1999.00024.x 11207556

[B8] Al-Younes H. M. Rudel T. Brinkmann V. Szczepek A. J. Meyer T. F. (2001). Low iron availability modulates the course of *Chlamydia pneumoniae* infection. Cell. Microbiol. 3, 427–437. 10.1046/j.1462-5822.2001.00125.x 11422085

[B9] Anderson J. E. Sparling P. F. Cornelissen C. N. (1994). Gonococcal transferrin-binding protein 2 facilitates but is not essential for transferrin utilization. J. Bacteriol. 176, 3162–3170. 10.1128/jb.176.11.3162-3170.1994 8195069 PMC205484

[B10] Andersson S. G. E. Kurland C. G. (1998). Reductive evolution of resident genomes. Trends Microbiol. 6, 263–268. 10.1016/S0966-842X(98)01312-2 9717214

[B11] Andrews N. C. (2000). Iron homeostasis: insights from genetics and animal models. Nat. Rev. Genet. 1, 208–217. 10.1038/35042073 11252750

[B12] Andrews S. C. Robinson A. K. Rodríguez-Quiñones F. (2003). Bacterial iron homeostasis. FEMS Microbiol. Rev. 27, 215–237. 10.1016/S0168-6445(03)00055-X 12829269

[B13] Bagg A. Neilands J. B. (1987). Ferric Uptake Regulation protein acts as a repressor, employing iron (ii) as a cofactor to bind the operator of an iron transport operon in *Escherichia coli*. Biochemistry 26, 5471–5477. 10.1021/bi00391a039 2823881

[B14] Bannantine J. P. Rockey D. D. (1999). Use of a primate model system to identify *Chlamydia trachomatis* protein antigens recognized uniquely in the context of infection. Microbiology 145, 2077–2085. 10.1099/13500872-145-8-2077 10463174

[B15] Barnewall R. E. Rikihisa Y. (1994). Abrogation of gamma interferon-induced inhibition of *Ehrlichia chaffeensis* infection in human monocytes with iron transferrin. Infect. Immun. 62, 4804–4810. 7927758 10.1128/iai.62.11.4804-4810.1994 PMC303190

[B16] Barnewall R. E. Ohashi N. Rikihisa Y. (1999). *Ehrlichia chaffeensis* and *E. sennetsu*, but Not the human granulocytic ehrlichiosis agent, colocalize with transferrin receptor and up-regulate transferrin receptor mRNA by activating iron-responsive protein 1. Infect. Immun. 67, 2258–2265. 10225882 10.1128/iai.67.5.2258-2265.1999 PMC115965

[B17] Barrile R. Kasendra M. Rossi-Paccani S. Merola M. Pizza M. Baldari C.. (2015). *Neisseria meningitidis* subverts the polarized organization and intracellular trafficking of host cells to cross the epithelial barrier. Cell. Microbiol. 17, 1365–1375. 10.1111/cmi.12439 25801707

[B18] Bearden S. W. Perry R. D. (1999). The Yfe system of *Yersinia pestis* transports iron and manganese and is required for full virulence of plague. Mol. Microbiol. 32, 403–414. 10.1046/j.1365-2958.1999.01360.x 10231495

[B19] Beatty W. L. Belanger T. A. Desai A. A. Morrison R. P. Byrne G. I. (1994). Tryptophan depletion as a mechanism of gamma interferon-mediated chlamydial persistence. Infect. Immun. 62, 3705–3711. 8063385 10.1128/iai.62.9.3705-3711.1994 PMC303021

[B20] Belland R. J. Nelson D. E. Virok D. Crane D. D. Hogan D. Sturdevant D.. (2003a). Transcriptome analysis of chlamydial growth during IFN-gamma-mediated persistence and reactivation. Proc. Natl. Acad. Sci. U.S.A. 100, 15971–15976. 10.1073/pnas.2535394100 14673075 PMC307677

[B21] Belland R. J. Zhong G. Crane D. D. Hogan D. Sturdevant D. Sharma J.. (2003b). Genomic transcriptional profiling of the developmental cycle of *Chlamydia trachomatis*. Proc. Natl. Acad. Sci. U.S.A. 100, 8478–8483. 10.1073/pnas.1331135100 12815105 PMC166254

[B22] Bellmann-Weiler R. Schroll A. Engl S. Nairz M. Talasz H. Seifert M.. (2013). Neutrophil gelatinase-associated lipocalin and interleukin-10 regulate intramacrophage *Chlamydia pneumoniae* replication by modulating intracellular iron homeostasis. Immunobiology 218, 969–978. 10.1016/j.imbio.2012.11.004 23317919 PMC3657155

[B23] Bennoun M. Devaux I. Beaumont C. Grandchamp B. Kahn A. Vaulont S. (2001). Lack of hepcidin gene expression and severe tissue iron overload in upstream stimulatory factor 2 (USF2) knockout mice. Proc. Natl. Acad. Sci. U.S.A. 98, 8780–8785. 10.1073/pnas.151179498 11447267 PMC37512

[B24] Berger T. Togawa A. Duncan G. S. Elia A. J. You-Ten A. Wakeham A.. (2006). Lipocalin 2-deficient mice exhibit increased sensitivity to *Escherichia coli* infection but not to ischemia-reperfusion injury. Proc. Natl. Acad. Sci. U.S.A. 103, 1834–1839. 10.1073/pnas.0510847103 16446425 PMC1413671

[B25] Boradia V. M. Malhotra H. Thakkar J. S. Tillu V. A. Vuppala B. Patil P.. (2014). *Mycobacterium tuberculosis* acquires iron by cell-surface sequestration and internalization of human holo-transferrin. Nat. Commun. 5:5730. 10.1038/ncomms5730 25163484

[B26] Boyd J. Oza M. N. Murphy J. R. (1990). Molecular cloning and DNA sequence analysis of a diphtheria tox iron-dependent regulatory element (dtxR) from *Corynebacterium diphtheriae*. Proc. Natl. Acad. Sci. U.S.A. 87, 5968–5972. 10.1073/pnas.87.15.5968 2116013 PMC54451

[B27] Briggs H. L. Pul N. Seshadri R. Wilson M. J. Tersteeg C. Russell-Lodrigue K. E.. (2008). Limited role for iron regulation in *Coxiella burnetii* Pathogenesis. Infect. Immun. 76, 2189–2201. 10.1128/IAI.01609-07 18316381 PMC2346684

[B28] Byrne G. I. Lehmann L. K. Landry G. J. (1986). Induction of tryptophan catabolism is the mechanism for gamma-interferon-mediated inhibition of intracellular *Chlamydia psittaci* replication in T24 cells. Infect. Immun. 53, 347–351. 3089936 10.1128/iai.53.2.347-351.1986 PMC260881

[B29] Cable H. Lloyd J. B. (1999). Cellular uptake and release of two contrasting iron chelators. J. Pharm. Pharmacol. 51, 131–134. 10.1211/0022357991772231 10217310

[B30] Calderwood S. B. Mekalanos J. J. (1987). Iron regulation of shiga-like toxin expression in *Escherichia coli* is mediated by the fur locus. J. Bacteriol. 169, 4759–4764. 10.1128/jb.169.10.4759-4764.1987 3308853 PMC213851

[B31] Carlson J. H. Wood H. Roshick C. Caldwell H. D. McClarty G. (2006). *In vivo* and *in vitro* studies of *Chlamydia trachomatis* TrpR:DNA interactions. Mol. Microbiol. 59, 1678–1691. 10.1111/j.1365-2958.2006.05045.x 16553875 PMC2808116

[B32] Carniel E. Rossi M. Fetherston J. D. Le S. Perry R. D. Ghigo J. (2001). Identification and characterization of the hemophore-dependent heme acquisition system of *Yersinia pestis*. Infect. Immun. 69, 6707–6717. 10.1128/IAI.69.11.6707-6717.2001 11598042 PMC100047

[B33] Carpenter B. M. Whitmire J. M. Merrell D. S. (2009). This is not your mother's repressor: the complex role of fur in pathogenesis. Infect. Immun. 77, 2590–2601. 10.1128/IAI.00116-09 19364842 PMC2708581

[B34] Carrano C. J. Raymond K. N. (1979). Ferric Iron Sequestering agents. 2. kinetics and mechanism of iron removal from transferrin by enterobactin and synthetic tricatechols. J. Am. Chem. Soc. 101, 5401–5404. 10.1021/ja00512a047

[B35] Case E. D. R. Akers J. C. Tan M. (2011). CT406 encodes a chlamydial ortholog of NrdR, a repressor of ribonucleotide reductase. J. Bacteriol. 193, 4396–4404. 10.1128/JB.00294-11 21725017 PMC3165505

[B36] Casey J. L. Koeller D. M. Ramin C. Klausner R. D. Harford J. B. (1989). Iron regulation of transferrin receptor mRNA levels requires iron-responsive elements and a rapid turnover determinant in the 3' untranslated region of the mRNA. EMBO J. 8, 3693–3699. 2583116 10.1002/j.1460-2075.1989.tb08544.x PMC402052

[B37] Caza M. Kronstad J. W. (2013). Shared and distinct mechanisms of iron acquisition by bacterial and fungal pathogens of humans. Front. Cell. Infect. Microbiol. 3:80. 10.3389/fcimb.2013.00080 24312900 PMC3832793

[B38] Chatfield C. H. Cianciotto N. P. (2007). The secreted pyomelanin pigment of *Legionella pneumophila* confers ferric reductase activity. Infect. Immun. 75, 4062–4070. 10.1128/IAI.00489-07 17548481 PMC1951983

[B39] Cheng Y. Zak O. Aisen P. Harrison S. C. Walz T. York N. (2004). Structure of the human transferrin receptor-transferrin complex albert einstein college of medicine. Cell 116, 565–576. 10.1016/S0092-8674(04)00130-8 14980223

[B40] Chung J. Chen C. Paw B. H. (2012). Heme metabolism and erythropoiesis. Curr. Opin. Hematol. 19, 156–162. 10.1097/MOH.0b013e328351c48b 22406824 PMC4086261

[B41] Clarke I. N. (2011). Evolution of *Chlamydia trachomatis*. Ann. N. Y. Acad. Sci. 1230, 11–18. 10.1111/j.1749-6632.2011.06194.x 22239534

[B42] Cockrell D. C. Long C. M. Robertson S. J. Shannon J. G. Miller E. Myers L. (2017). Robust growth of a virulent phase II *Coxiella burnetii* in bone marrow-derived murine macrophages. PLoS ONE 12:173528 10.1371/journal.pone.0173528 PMC5344453 28278296

[B43] Cohen M. Britigan B. French M. Bean K. (1987). Preliminary observations on lactoferrin secretion in human vaginal mucus: variation during the menstrual cycle, evidence of hromonal regulation, and implications for infection with *Neisseria gonorrhoeae*. Am. J. Obstet. Gynecol. 157, 1122–1125. 10.1016/S0002-9378(87)80274-0 3120589

[B44] Cornelissen C. N. A. U. Biswas G. D. Tsai J. Paruchuri D. K. Thompson S. A. Sparling P. F. (1992). Gonococcal transferrin-binding protein 1 is required for transferrin utilization and is homologous to tonB-dependent outer membrane receptors. J. Bacteriol. 174, 5788–5797. 10.1128/jb.174.18.5788-5797.1992 1325963 PMC207106

[B45] Crichton R. R. (ed.). (2016). Cellular iron uptake and export in mammals, in Iron Metabolism: From Molecular Mechanisms to Clinical Consequences (Chichester: John Wiley & Sons, Inc.), 364–428.

[B46] Crossley R. A. Gaskin D. J. H. Holmes K. Mulholland F. Wells J. M. Kelly D. J.. (2007). Riboflavin biosynthesis is associated with assimilatory ferric reduction and iron acquisition by *Campylobacter jejuni*. Appl. Environ. Microbiol. 73, 7819–7825. 10.1128/AEM.01919-07 17965203 PMC2168145

[B47] Dautry-varsat A. Ciechanover A. Lodish H. F. (1983). pH and the recycling of transferrin during receptor- mediated endocytosis. Proc. Natl. Acad. Sci. U.S.A. 80, 2258–2262. 10.1073/pnas.80.8.2258 6300903 PMC393798

[B48] de Lorenzo V. Wee S. Herrero M. Neilands J. B. (1987). Operator sequences of the aerobactin operon of plasmid ColV-K30 binding the ferric uptake regulation (fur) repressor. J. Bacteriol. 169, 2624–2630. 10.1128/jb.169.6.2624-2630.1987 3294800 PMC212138

[B49] Derré I. Pypaert M. Dautry-Varsat A. Agaisse H. (2007). RNAi screen in Drosophila cells reveals the involvement of the tom complex in Chlamydia infection. PLoS Pathog. 3:30155. 10.1371/journal.ppat.0030155 17967059 PMC2042019

[B50] Dill B. D. Raulston J. E. (2007). Examination of an inducible expression system for limiting iron availability during *Chlamydia trachomatis* infection. Microbes Infect. 9, 947–953. 10.1016/j.micinf.2007.03.017 17544798 PMC2083192

[B51] Dill B. D. Dessus-Babus S. Raulston J. E. (2009). Identification of iron-responsive proteins expressed by *Chlamydia trachomatis* reticulate bodies during intracellular growth. Microbiology 155, 210–219. 10.1099/mic.0.022731-0 19118361

[B52] Donati M. Huot-Creasy H. Humphrys M. Paolo M. Di Francesco A. Myersb G. S. A. (2014). Genome sequence of *Chlamydia suis* MD56, isolated from the conjunctiva of a weaned piglet. Genome Announc. 2:2147. 10.1128/genomeA.00425-14 24812227 PMC4014695

[B53] Donovan A. Brownlie A. Zhou Y. Shepard J. Pratt S. J. Moynihan J.. (2000). Positional cloning of zebrafish ferroportin1 identifies a conserved vertebrate iron exporter. Nature 403, 776–781. 10.1038/35001596 10693807

[B54] Doyle C. K. Zhang X. Popov V. L. McBride J. W. (2005). An immunoreactive 38-kilodalton protein of *Ehrlichia canis* shares structural homology and iron-binding capacity with the ferric ion-binding protein family. Infect. Immun. 73, 62–69. 10.1128/IAI.73.1.62-69.2005 15618141 PMC538948

[B55] Drakesmith H. Prentice A. M. (2012). Hepcidin and the iron-infection axis. Science 338, 768–772. 10.1126/science.1224577 23139325

[B56] Ellison D. W. Clark T. R. Sturdevant D. E. Virtaneva K. Hackstadt T. (2009). Limited transcriptional responses of *Rickettsia rickettsii* exposed to environmental stimuli. PLoS ONE 4:5612. 10.1371/journal.pone.0005612 19440298 PMC2680988

[B57] Ellison D. W. Clark T. R. Sturdevant D. E. Virtaneva K. Porcella S. F. Hackstadt T. (2008). Genomic comparison of virulent *Rickettsia rickettsii* Sheila Smith and avirulent *Rickettsia rickettsii* Iowa. Infect. Immun. 76, 542–550. 10.1128/IAI.00952-07 18025092 PMC2223442

[B58] Ernst J. F. Bennert R. L. Rothfield L. I. (1978). Constitutive expression of the iron-enterochelin and ferrichrome uptake systems in a mutant strain of *Salmonella typhimurium*. J. Bacteriol. 135, 928–934. 151097 10.1128/jb.135.3.928-934.1978 PMC222466

[B59] Escolar L. de Lorenzo V. Perez-Martin J. (1997). Metalloregulation *in vitro* of the aerobactin promoter of *Escherichia coli* by the Fur (ferric uptake regulation) protein. Mol. Microbiol. 26, 799–808. 10.1046/j.1365-2958.1997.6211987.x 9427409

[B60] Escolar L. Pérez-Martín J. De Lorenzo V. (1998). Coordinated repression *in vitro* of the divergent fepA-fes promoters of *Escherichia coli* by the iron uptake regulation (Fur) protein. J. Bacteriol. 180, 2579–2582. 9573216 10.1128/jb.180.9.2579-2582.1998 PMC107206

[B61] Fecker L. Braun V. (1983). Cloning and expression of the fhu genes involved in iron(III)-hydroxamate uptake by *Escherichia coli*. J. Bacteriol. 156, 1301–1314. 6315685 10.1128/jb.156.3.1301-1314.1983 PMC217981

[B62] Fleming M. D. Romano M. A. Su M. A. Garrick L. M. Garrick M. D. Andrews N. C. (1998). Nramp 2 is mutated in the anemic Belgrade (b) rat : Evidence of a role for Nramp2 in endosomal iron transport. Proc. Natl. Acad. Sci. U.S.A. 95, 1148–1153. 10.1073/pnas.95.3.1148 9448300 PMC18702

[B63] Fleming M. Trenor C. III. Su M. Foernzler D. Beier D. Dietrich W.. (1997). Microcytic anaemia mice have a mutation in Nramp2, a candidate iron transporter gene. Nat. Genet. 16, 383–386. 10.1038/ng0897-383 9241278

[B64] Flo T. H. T. Smith K. D. K. Sato S. Rodriguez D. J. D. J. Holmes M. A. Strong R. K.. (2004). Lipocalin 2 mediates an innate immune response to bacterial infection by sequestrating iron. Nature 432, 917–921. 10.1038/nature03104 15531878

[B65] Freidank H. M. Billing H. Wiedmann-Al-Ahmad M. (2001). Influence of iron restriction on *Chlamydia pneumoniae* and *C*. trachomatis. J. Med. Microbiol. 50, 223–227. 10.1099/0022-1317-50-3-223 11232766

[B66] Frey P. A. Reed G. H. (2012). The ubiquity of iron. ACS Chem. Biol. 7, 1477–1481. 10.1021/cb300323q 22845493

[B67] Gaballa A. Helmann J. D. (1998). Identification of a zinc-specific metalloregulatory protein, zur, controlling zinc transport operons in *Bacillus subtilis*. J. Bacteriol. 180, 5815–5821. 9811636 10.1128/jb.180.22.5815-5821.1998 PMC107652

[B68] Gancz H. Censini S. Merrell D. S. (2006). Iron and pH homeostasis intersect at the level of fur regulation in the gastric pathogen *Helicobacter pylori*. Infect. Immun. 74, 602–614. 10.1128/IAI.74.1.602-614.2006 16369017 PMC1346641

[B69] Ganz T. Nemeth E. (2015). Iron homeostasis in host defence and inflammation. Nat. Rev. Immunol. 15, 500–510. 10.1038/nri3863 26160612 PMC4801113

[B70] Glickstein H. El R. Ben M. Cabantchik Z. I. (2005). Intracellular labile iron pools as direct targets of iron chelators: a fluorescence study of chelator action in living cells. Blood 106, 3242–3251. 10.1182/blood-2005-02-0460 16020512

[B71] Goellner S. Schubert E. Liebler-Tenorio E. Hotzel H. Saluz H. P. Sachse K. (2006). Transcriptional response patterns of *Chlamydophila psittaci* in different *in vitro* models of persistent infection. Infect. Immun. 74, 4801–4808. 10.1128/IAI.01487-05 16861668 PMC1539575

[B72] Gold B. Rodriguez G. M. Salvatore A. Marras E. Pentecost M. Smith I. (2001). The *Mycobacterium tuberculosis* IdeR is a dual functional regulator that controls transcription of genes involved in iron acquisition, iron storage and survival in macrophages. Mol. Microbiol. 42, 851–865. 10.1046/j.1365-2958.2001.02684.x 11722747

[B73] Görke B. Reinhardt J. Rak B. (2005). Activity of Lac repressor anchored to the *Escherichia coli* inner membrane. Nucleic Acids Res. 33, 2504–2511. 10.1093/nar/gki549 15867195 PMC1088070

[B74] Gottesman S. Masse E. (2002). A small RNA regulates the expression of genes involved in iron metabolism in *Escherichia coli*. Proc. Natl. Acad. Sci. U.S.A. 99, 4620–4625. 10.1073/pnas.032066599 11917098 PMC123697

[B75] Gresock M. G. Kastead K. A. Postle K. (2015). From homodimer to heterodimer and back: elucidating the TonB energy transduction cycle. J. Bacteriol. 197, 3433–3445. 10.1128/JB.00484-15 26283773 PMC4621073

[B76] Gresock M. G. Savenkova M. I. Larsen R. A. Ollis A. A. Postle K. (2011). Death of the TonB shuttle hypothesis. Front. Microbiol. 2:206. 10.3389/fmicb.2011.00206 22016747 PMC3191458

[B77] Grinblat-huse V. Drabek E. F. Creasy H. H. Daugherty S. C. Jones K. M. Santana-cruz I.. (2011). Genome sequences of the zoonotic pathogens *Chlamydia psittaci*. J. Bacteriol. 193, 4039–4040. 10.1128/JB.05277-11 21622741 PMC3147492

[B78] Gunshin H. Mackenzie B. Berger U. V. Gunshin Y. Romero M. F. Boron W. F.. (1997). Cloning and characterization of a mammalian proton-coupled metal-ion transporter. Nature 388, 482–488. 10.1038/41343 9242408

[B79] Halaas O. Steigedal M. Haug M. Awuh J. A. Ryan L. Brech A.. (2010). Intracellular *Mycobacterium avium* intersect transferrin in the Rab11^+^ recycling endocytic pathway and avoid lipocalin 2 trafficking to the lysosomal pathway. J. Infect. Dis. 201, 783–792. 10.1086/650493 20121435 PMC2862295

[B80] Hamza I. Chauhan S. Hassett R. O'Brian M. R. (1998). The bacterial irr protein is required for coordination of heme biosynthesis with iron availability. J. Biol. Chem. 273, 21669–21674. 9705301 10.1074/jbc.273.34.21669

[B81] Hamza I. Qi Z. King N. D. O'Brian M. R. (2000). Fur-independent regulation of iron metabolism by Irr in *Bradyrhizobium japonicum*. Microbiology 146, 669–676. 10.1099/00221287-146-3-669 10746770

[B82] Hantke K. (1981). Regulation of ferric iron transport in *Escherichia coli* K12: isolation of a constitutive mutant. Mol. Gen. Genet. 182, 288–292. 10.1007/BF00269672 7026976

[B83] Hantke K. (1982). Negative control of iron uptake systems in *Escherichia coli*. FEMS Microbiol. Lett. 15, 83–86. 10.1111/j.1574-6968.1982.tb00043.x

[B84] Hantke K. (2001). Iron and metal regulation in bacteria. Curr. Opin. Microbiol. 4, 172–177. 10.1016/S1369-5274(00)00184-3 11282473

[B85] Harding C. Heuser J. Stahl P. (1983). Receptor-mediated endocytosis of transferrin and recycling of the transferrin receptor in rat reticulocytes biochemical approaches to transferrin. J. Cell Biol. 97, 329–339. 10.1083/jcb.97.2.329 6309857 PMC2112509

[B86] Harrison P. M. Arosio P. (1996). The ferritins: molecular properties, iron storage function and cellular regulation. Biochim. Biophys. Acta 1275, 161–203. 10.1016/0005-2728(96)00022-9 8695634

[B87] Hellman N. E. Gitlin J. D. (2002). Ceruloplasmin metabolsim and function. Annu. Rev. Nutr. 22, 439–458. 10.1146/annurev.nutr.22.012502.114457 12055353

[B88] Hentze M. W. Muckenthaler M. U. Andrews N. C. (2004). Balancing acts : molecular control of mammalian iron metabolism. Cell 117, 285–297. 10.1016/S0092-8674(04)00343-5 15109490

[B89] Hentze M. W. Muckenthaler M. U. Galy B. Camaschella C. (2010). Two to tango: regulation of mammalian iron metabolism. Cell 142, 24–38. 10.1016/j.cell.2010.06.028 20603012

[B90] Hohle T. H. O'Brian M. R. (2010). Transcriptional control of the *Bradyrhizobium japonicum* irr gene requires repression by fur and antirepression by irr. J. Biol. Chem. 285, 26074–26080. 10.1074/jbc.M110.145979 20573962 PMC2924008

[B91] Hohle T. H. O'Brian M. R. (2016). Metal-specific control of gene expression mediated by *Bradyrhizobium japonicum* mur and *Escherichia coli* fur is determined by the cellular context. Mol. Microbiol. 101, 152–166. 10.1111/mmi.13381 26998998 PMC4925281

[B92] Humphrys M. S. Creasy T. Sun Y. Shetty A. C. Chibucos M. C. Drabek E. F.. (2013). Simultaneous transcriptional profiling of bacteria and their host cells. PLoS ONE 8:80597. 10.1371/journal.pone.0080597 24324615 PMC3851178

[B93] Kalman S. Mitchell W. Marathe R. Lammel C. Fan J. Hyman R. W.. (1999). Comparative genomes of *Chlamydia pneumoniae* and *C*. trachomatis. Nat. Genet. 21, 385–389. 10.1038/7716 10192388

[B94] Kammler M. Schon C. Hantke K. (1993). Characterization of the ferrous iron uptake system of *Escherichia coli*. J. Bacteriol. 175, 6212–6219. 10.1128/jb.175.19.6212-6219.1993 8407793 PMC206716

[B95] Keberle H. (1964). The biochemistry of desferrioxamine and its relation to iron metabolism. Ann. N.Y. Acad. Sci. 119, 758–768. 10.1111/j.1749-6632.1965.tb54077.x 14219455

[B96] Kelver M. E. Kaul A. Nowicki B. Findley W. E. Hutchens T. W. Nagamani M. (1996). Estrogen regulation of lactoferrin expression in human endometrium. Am. J. Reprod. Immunol. 36, 243–247. 10.1111/j.1600-0897.1996.tb00171.x 8955499

[B97] Kemege K. E. Hickey J. M. Lovell S. Battaile K. P. Zhang Y. Hefty P. S. (2011). Ab initio structural modeling of and experimental validation for *Chlamydia trachomatis* protein CT296 reveal structural similarity to Fe(II) 2-oxoglutarate-dependent enzymes. J. Bacteriol. 193, 6517–6528. 10.1128/JB.05488-11 21965559 PMC3232896

[B98] Kim H. J. Khalimonchuk O. Smith P. M. Winge D. R. (2012). Structure, function, and assembly of heme centers in mitochondrial respiratory complexes. Biochim. Biophys. Acta 1823, 1604–1616. 10.1016/j.bbamcr.2012.04.008 22554985 PMC3601904

[B99] Koo I. C. Walthers D. Hefty P. S. Kenney L. J. Stephens R. S. (2006). ChxR is a transcriptional activator in Chlamydia. Proc. Natl. Acad. Sci. U.S.A. 103, 750–755. 10.1073/pnas.0509690103 16407127 PMC1325966

[B100] Kosman D. J. (2003). Molecular mechanisms of iron uptake in fungi. Mol. Microbiol. 47, 1185–1197. 10.1046/j.1365-2958.2003.03368.x 12603727

[B101] Kumar R. Lovell S. Matsumura H. Battaile K. P. Moenne-Loccoz P. Rivera M. (2013). The hemophore hasa from *Yersinia pestis* (HasAyp) coordinates hemin with a single residue, Tyr75, and with minimal conformational change. Biochemistry 52, 2705–2707. 10.1021/bi400280z 23578210 PMC4486319

[B102] Kunkle C. A. Schmitt M. P. (2005). Analysis of a DtxR-regulated iron transport and siderophore biosynthesis gene cluster in *Corynebacterium diphtheriae*. J. Bacteriol. 187, 422–433. 10.1128/JB.187.2.422-433.2005 15629913 PMC543566

[B103] LaRue R. W. Dill B. D. Giles D. K. Whittimore J. D. Raulston J. E. (2007). Chlamydial Hsp60-2 is iron responsive in *Chlamydia trachomatis* serovar E-infected human endometrial epithelial cells *in vitro*. Infect. Immun. 75, 2374–2380. 10.1128/IAI.01465-06 17307941 PMC1865735

[B104] Lawson D. M. Treffry A. Artyrniuk P. J. Harrison P. M. Yewdall S. J. Luzzago A.. (1989). Identification of the ferroxidase centre in ferritin. FEBS Lett. 254, 207–210. 10.1016/0014-5793(89)81040-3 2776883

[B105] Lee J. H. Wang T. Ault K. Liu J. Schmitt M. P. Holmes R. K. (1997). Identification and characterization of three new promoter/operators from *Corynebacterium diphtheriae* that are regulated by the diphtheria toxin repressor (DtxR) and iron. Infect. Immun. 65, 4273–4280. 9317037 10.1128/iai.65.10.4273-4280.1997 PMC175613

[B106] Lee J. W. Helmann J. D. (2007). Functional specialization within the fur family of metalloregulators. Biometals 20, 485–499. 10.1007/s10534-006-9070-7 17216355

[B107] Lee Y. Deka R. K. Michael V. Radolf J. D. Hasemann C. A. (1999). *Treponema pallidum* TroA is a periplasmic zinc-binding protein with a helical backbone. Nat. Struct. Biol. 6, 628–633. 10.1038/10677 10404217

[B108] Lefèvre J. Delepelaire P. Delepierre M. Izadi-pruneyre N. (2008). Modulation by substrates of the interaction between the HasR outer membrane receptor and its specific. J. Mol. Biol. 378, 840–851. 10.1016/j.jmb.2008.03.044 18402979

[B109] Lewis M. E. Belland R. J. AbdelRahman Y. M. Beatty W. L. Aiyar A. A. Zea A. H.. (2014). Morphologic and molecular evaluation of *Chlamydia trachomatis* growth in human endocervix reveals distinct growth patterns. Front. Cell. Infect. Microbiol. 4:71. 10.3389/fcimb.2014.00071 24959423 PMC4050528

[B110] Lipinski A. R. Heymann J. Meissner C. Karlas A. Brinkmann V. Meyer T. F.. (2009). Rab6 and Rab11 regulate *Chlamydia trachomatis* development and golgin-84-dependent golgi fragmentation. PLoS Pathog. 5:e1000615. 10.1371/journal.ppat.1000615 19816566 PMC2752117

[B111] Lloyd J. B. Cable H. Rice-Evans C. (1991). Evidence that desferrioxamine cannot enter cells by passive diffusion. Biochem. Pharmacol. 41, 1361–1363. 10.1016/0006-2952(91)90109-I 2018567

[B112] Maass M. Essig A. Marre R. Henkel W. (1993). Growth in serum-free medium improves isolation of *Chlamydia pneumoniae*. J. Clin. Microbiol. 31, 3050–3052. 8263198 10.1128/jcm.31.11.3050-3052.1993 PMC266211

[B113] Marx J. J. M. (2002). Iron and infection: competition between host and microbes for a precious element. Best Pract. Res. Clin. Haematol. 15, 411–426. 10.1053/beha.2002.0001 12401315

[B114] Matsumoto A. Bessho H. Uehira K. Suda T. (1991). Morphological studies of the association of mitochondria with chlamydial inclusions and the fusion of chlamydial inclusions. J. Electron. Microsc. 40, 356–363. 10.1093/oxfordjournals.jmicro.a050908 1666645

[B115] Mäurer A. P. Mehlitz A. Mollenkopf H. J. Meyer T. F. (2007). Gene expression profiles of *Chlamydophila pneumoniae* during the developmental cycle and iron depletion-mediated persistence. PLoS Pathog. 3, 0752–0769. 10.1371/journal.ppat.0030083 17590080 PMC1894823

[B116] Mayle K. M. Le A. M. Kamei D. T. (2012). The intracellular trafficking pathway of transferrin. Biochim. Biophys. Acta 1820, 264–281. 10.1016/j.bbagen.2011.09.009 21968002 PMC3288267

[B117] McKie A. T. Barrow D. Latunde-dada G. O. Rolfs A. Sager G. Mudaly E.. (2001). An iron-regulated ferric reductase associated with the absorption of dietary iron. Science 291, 1755–1759. 10.1126/science.1057206 11230685

[B118] Mehi O. Bogos B. Csorgo B. Pal F. Nyerges A. Papp B.. (2014). Perturbation of iron homeostasis promotes the evolution of antibiotic resistance. Mol. Biol. Evol. 31, 2793–2804. 10.1093/molbev/msu223 25063442 PMC4166929

[B119] Mellouk N. Enninga J. (2016). Cytosolic access of intracellular bacterial pathogens: the shigella paradigm. Front. Cell. Infect. Microbiol. 6:35. 10.3389/fcimb.2016.00035 27092296 PMC4820437

[B120] Mertens K. Samuel J. E. (2012). Defense mechanisms against oxidative stress in *Coxiella burnetii*: adaptation to a unique intracellular niche, in Coxiella burnetii: Recent Advances and New Perspectives in Research of the Q Fever Bacterium, eds Toman R. Heinzen R. A. Samuel J. E. Mege J. L. (Dordrecht: Springer Netherlands), 39–63. 10.1007/978-94-007-4315-1_3 22711626

[B121] Mietzner T. A. Luginbuhl G. H. Sandstrom E. Morse S. A. (1984). Identification of an iron-regulated 37,000-dalton protein in the cell envelope of *Neisseria gonorrhoeae*. Infect. Immun. 45, 410–416. 6430806 10.1128/iai.45.2.410-416.1984 PMC263238

[B122] Miller J. D. Sal M. S. Schell M. Whittimore J. D. Raulston J. E. (2009). *Chlamydia trachomatis* YtgA is an iron-binding periplasmic protein induced by iron restriction. Microbiology 155, 2884–2894. 10.1099/mic.0.030247-0 19556290 PMC2888172

[B123] Mojica S. Creasy H. H. Daugherty S. Read T. D. Kim T. Kaltenboeck B.. (2011). Genome sequence of the obligate intracellular animal pathogen *Chlamydia pecorum* E58. J. Bacteriol. 193, 9193. 10.1128/JB.00454-11 21571992 PMC3133325

[B124] Moore E. R. Ouellette S. P. (2014). Reconceptualizing the chlamydial inclusion as a pathogen-specified parasitic organelle: an expanded role for Inc proteins. Front. Cell. Infecit. Microbiol. 4:157. 10.3389/fcimb.2014.00157 25401095 PMC4215707

[B125] Mukhopadhyay S. Miller R. D. Sullivan E. D. Theodoropoulos C. Mathews S. A. Timms P.. (2006). Protein expression profiles of *Chlamydia pneumoniae* in models of persistence versus those of heat shock stress response. Infect. Immun. 74, 3853–3863. 10.1128/IAI.02104-05 16790757 PMC1489704

[B126] Murphy J. R. Pappenheimer A. M. Jr. Tayart de Borms S. (1974). Synthesis of diphtheria tox-gene products in *Eacherichia coli* Extracts. Proc. Natl. Acad. Sci. U.S.A. 71, 11–15. 10.1073/pnas.71.1.11 4204202 PMC387921

[B127] Nairz M. Haschka D. Demetz E. Weiss G. (2014). Iron at the interface of immunity and infection. Front. Pharmacol. 5:152. 10.3389/fphar.2014.00152 25076907 PMC4100575

[B128] Nairz M. Theurl I. Schroll A. Theurl M. Fritsche G. Lindner E.. (2009). Absence of functional Hfe protects mice from invasive *Salmonella enterica* serovar typhimurium infection via induction of lipocalin-2. Blood 114, 3642–3651. 10.1182/blood-2009-05-223354 19700664 PMC2766679

[B129] Nemeth E. Tuttle M. S. Powelson J. Vaughn M. B. Donovan A. Ward D. M.. (2004). Hepcidin regulates cellular iron efflux by binding to ferroportin and inducing its internalization. Science 306, 2090–2093. 10.1126/science.1104742 15514116

[B130] Neufeld E. J. (2006). Oral chelators deferasirox and defefiprone for transfusional iron overload in thalassen a major: new data, new questions. Blood 107, 3436–3441. 10.1182/blood-2006-02-002394 16627763 PMC1895765

[B131] Nienaber A. Hennecke H. Fischer H. M. (2001). Discovery of a haem uptake system in the soil bacterium *Bradyrhizobium japonicum*. Mol. Microbiol. 41, 787–800. 10.1046/j.1365-2958.2001.02555.x 11532144

[B132] Noinaj N. Buchanan S. K. Cornelissen C. N. (2012a). The transferrin—iron import system from pathogenic Neisseria species. Mol. Microbiol. 86, 246–257. 10.1111/mmi.12002 22957710 PMC3468669

[B133] Noinaj N. Easley N. C. Oke M. Mizuno N. Gumbart J. Boura E.. (2012b). Structural basis for iron piracy by pathogenic Neisseria. Nature 482, 53–58. 10.1038/nature10823 22327295 PMC3292680

[B134] Nystrom-Rosander C. Lindh U. Ilback N.-G. Hjelm E. Thelin S. Lindqvist O.. (2003). Interactions between *Chlamydia pneumoniae* and trace elements: a possible link to aortic valve sclerosis. Biol. Trace Elem. Res. 91, 97–110. 10.1385/BTER:91:2:97 12719605

[B135] Oexle H. Gnaiger E. Weiss G. (1999). Iron-dependent changes in cellular energy metabolism: influence on citric acid cycle and oxidative phosphorylation. Biochim. Biophys. Acta 1413, 99–107. 10.1016/S0005-2728(99)00088-2 10556622

[B136] Ohgami R. S. Campagna D. R. Greer E. L. Antiochos B. McDonald A. Chen J.. (2005). Identification of a ferrireductase required for efficient transferrin-dependent iron uptake in erythroid cells. Nat. Genet. 37, 1264–1269. 10.1038/ng1658 16227996 PMC2156108

[B137] Ojeda J. F. Martinson D. A. Menscher E. A. Roop R. M. (2012). The bhuQ gene encodes a heme oxygenase that contributes to the ability of *Brucella abortus* 2308 to use heme as an iron source and is regulated by Irr. J. Bacteriol. 194, 4052–4058. 10.1128/JB.00367-12 22636783 PMC3416563

[B138] Osaki S. Johnson D. A. Frieden E. (1966). The Possible Significance of the ferrous oxidase activity of ceruloplasmin in normal human serum. J. Biol. Chem. 241, 2746–2751. 5912351

[B139] Ostberg K. L. DeRocco A. J. Mistry S. D. Dickinson M. K. Cornelissen C. N. (2013). Conserved regions of gonococcal TbpB are critical for surface exposure and transferrin iron utilization. Infect. Immun. 81, 3442–3450. 10.1128/IAI.00280-13 23836816 PMC3754215

[B140] Ouellette S. P. Carabeo R. A. (2010). A functional slow recycling pathway of transferrin is required for growth of Chlamydia. Front. Microbiol. 1:112. 10.3389/fmicb.2010.00112 21607082 PMC3095398

[B141] Ouellette S. P. Dorsey F. C. Moshiach S. Cleveland J. L. Carabeo R. A. (2011). Chlamydia species-dependent differences in the growth requirement for lysosomes. PLoS ONE 6:16783. 10.1371/journal.pone.0016783 21408144 PMC3050816

[B142] Ouellette S. P. Hatch T. P. AbdelRahman Y. M. Rose L. A. Belland R. J. Byrne G. I. (2006). Global transcriptional upregulation in the absence of increased translation in Chlamydia during IFNγ-mediated host cell tryptophan starvation. Mol. Microbiol. 62, 1387–1401. 10.1111/j.1365-2958.2006.05465.x 17059564

[B143] Outten C. E. Tobin D. A. Penner-Hahn J. E. O'Halloran T. V. O. (2001). Characterization of the metal receptor sites in *Escherichia coli* zur, an ultrasensitive Zinc(II) metalloregulatory protein. Biochemistry 40, 10417–10423. 10.1021/bi0155448 11523983

[B144] Ozenberger B. A. Nahlik M. S. McIntosh M. A. (1987). Genetic organization of multiple fep genes encoding ferric enterobactin transport functions in *Escherichia coli*. J. Bacteriol. 169, 3638–3646. 10.1128/jb.169.8.3638-3646.1987 2956250 PMC212444

[B145] Paradkar P. N. Domenico I. De Durchfort N. Zohn I. Kaplan J. Ward D. M. (2008). Iron depletion limits intracellular bacterial growth in macrophages. Blood 112, 866–874. 10.1182/blood-2007-12-126854 18369153 PMC2481528

[B146] Parrow N. L. Abbott J. Lockwood A. R. Battisti J. M. Minnick M. F. (2009). Function, regulation, and transcriptional organization of the hemin utilization locus of *Bartonella quintana*. Infect. Immun. 77, 307–316. 10.1128/IAI.01194-08 18981245 PMC2612243

[B147] Patzer S. I. Hantke K. (2001). Dual repression by Fe(2+)-Fur and Mn(2+)-MntR of the mntH Gene, encoding an NRAMP-like Mn(2+) transporter in *Escherichia coli*. J. Bacteriol. 183, 4806–4813. 10.1128/JB.183.16.4806-4813.2001 11466284 PMC99535

[B148] Persson H. L. Yu Z. Tirosh O. Eaton J. W. Brunk U. T. (2003). Prevention of oxidant-induced cell death by lysosomotropic iron chelators. Free Radic. Biol. Med. 34, 1295–1305. 10.1016/S0891-5849(03)00106-0 12726917

[B149] Phillips Campbell R. Kintner J. Whittimore J. Schoborg R. V. (2012). Chlamydia muridarum enters a viable but non-infectious state in amoxicillin-treated BALB/c mice. Microbes Infect. 14, 1177–1185. 10.1016/j.micinf.2012.07.017 22943883 PMC3654801

[B150] Pieper R. Fisher C. R. Suh M. J. Huang S. T. Parmar P. Payne S. M. (2013). Analysis of the proteome of intracellular *Shigella flexneri* reveals pathways important for intracellular growth. Infect. Immun. 81, 4635–4648. 10.1128/IAI.00975-13 24101689 PMC3837999

[B151] Pierce J. R. Pickett C. L. Earhart C. F. (1983). Two fep genes are required for Ferrienterochelin Uptake in *Escherichia coli* K-12. J. Bacteriol. 155, 330–336. 6223021 10.1128/jb.155.1.330-336.1983 PMC217684

[B152] Pigeon C. Ilyin G. Courselaud B. Leroyer P. Turlin B. Brissot P.. (2001). A new mouse liver-specific gene, encoding a protein homologous to human antimicrobial peptide hepcidin, is overexpressed during iron overload. J. Biol. Chem. 276, 7811–7819. 10.1074/jbc.M008923200 11113132

[B153] Pohl E. Haller J. C. Mijovilovich A. Meyer-Klaucke W. Garman E. Vasil M. L. (2003). Architecture of a protein central to iron homeostasis: crystal structure and spectroscopic analysis of the ferric uptake regulator. Mol. Microbiol. 47, 903–915. 10.1046/j.1365-2958.2003.03337.x 12581348

[B154] Pohl E. Holmes R. K. Hol W. G. J. (1999). Crystal Structure of a cobalt-activated diphtheria toxin repressor-DNA complex reveals a metal-binding SH3-like domain. J. Mol. Biol. 292, 653–667. 10.1006/jmbi.1999.3073 10497029

[B155] Posey J. E. Gherardini F. C. (2000). Lack of a role for iron in the Lyme disease pathogen. Science 288, 1651–1653. 10.1126/science.288.5471.1651 10834845

[B156] Posey J. E. Hardham J. M. Norris S. J. Gherardini F. C. (1999). Characterization of a manganese-dependent regulatory protein, TroR, from *Treponema pallidum*. Proc. Natl. Acad. Sci. U.S.A. 96, 10887–10892. 10.1073/pnas.96.19.10887 10485921 PMC17978

[B157] Pospischil A. Borel N. Chowdhury E. H. Guscetti F. (2009). Aberrant chlamydial developmental forms in the gastrointestinal tract of pigs spontaneously and experimentally infected with *Chlamydia suis*. Vet. Microbiol. 135, 147–156. 10.1016/j.vetmic.2008.09.035 18950970

[B158] Puri S. O'Brian M. R. (2006). The hmuQ and hmuD genes from *Bradyrhizobium japonicum* encode heme-degrading enzymes. J. Bacteriol. 188, 6476–6482. 10.1128/JB.00737-06 16952937 PMC1595471

[B159] Qi Z. O'Brian M. R. (2002). Interaction between the bacterial iron response regulator and ferrochelatase mediates genetic control of heme biosynthesis. Mol. Cell 9, 155–162. 10.1016/S1097-2765(01)00431-2 11804594

[B160] Qi Z. Hamza I. O'Brian M. R. (1999). Heme is an effector molecule for iron-dependent degradation of the bacterial iron response regulator (Irr) protein. Proc. Natl. Acad. Sci. U.S.A. 96, 13056–13061. 10.1073/pnas.96.23.13056 10557272 PMC23899

[B161] Que Q. Helmann J. D. (2000). Manganese homeostasis in *Bacillus subtilis* is regulated by MntR, a bifunctional regulator related to the diphtheria toxin repressor family of proteins. Mol. Microbiol. 35, 1454–1468. 10.1046/j.1365-2958.2000.01811.x 10760146

[B162] Ratledge C. Dover L. G. (2000). Iron metabolism in pathogenic bacteria. Annu. Rev. Microbiol. 54, 881–941. 10.1146/annurev.micro.54.1.881 11018148

[B163] Rau A. Wyllie S. Whittimore J. Raulston J. E. (2005). Identification of *Chlamydia trachomatis* genomic sequences recognized by chlamydial divalent cation-dependent regulator A (DcrA). J. Bacteriol. 187, 443–448. 10.1128/JB.187.2.443-448.2005 15629915 PMC543534

[B164] Raulston J. E. (1997). Response of *Chlamydia trachomatis* serovar E to iron restriction *in vitro* and evidence for iron-regulated chlamydial proteins. Infect. Immun. 65, 4539–4547. 9353031 10.1128/iai.65.11.4539-4547.1997 PMC175652

[B165] Raulston J. E. Miller J. D. Davis C. H. Schell M. Baldwin A. Ferguson K.. (2007). Identification of an iron-responsive protein that is antigenic in patients with *Chlamydia trachomatis* genital infections. FEMS Immunol. Med. Microbiol. 51, 569–576. 10.1111/j.1574-695X.2007.00336.x 17991015

[B166] Read T. D. Brunham R. C. Shen C. Gill S. R. Heidelberg J. F. White O.. (2000). Genome sequences of *Chlamydia trachomatis* MoPn and *Chlamydia pneumoniae* AR39. Nucleic Acids Res. 28, 1397–1406. 10.1093/nar/28.6.1397 10684935 PMC111046

[B167] Read T. D. Myers G. S. A. Brunham R. C. Nelson W. C. Paulsen I. T. Heidelberg J.. (2003). Genome sequence of *Chlamydophila caviae* (*Chlamydia psittaci* GPIC): examining the role of niche-specific genes in the evolution of the Chlamydiaceae. Nucleic Acids Res. 31, 2134–2147. 10.1093/nar/gkg321 12682364 PMC153749

[B168] Richardson D. Ponka P. Baker E. (1994). The effect of the iron(III) chelator, desferrioxamine, on iron and transferrin. Cancer Res. 54, 685–689. 8306330

[B169] Rohde K. H. Dyer D. W. (2004). Analysis of haptoglobin and hemoglobin-haptoglobin interactions with the *Neisseria meningitidis* TonB-dependent receptor HpuAB by flow cytometry. Infect. Immun. 72, 2494–2506. 10.1128/IAI.72.5.2494-2506.2004 15102756 PMC387877

[B170] Rosario C. J. Tan M. (2012). The early gene product EUO is a transcriptional repressor that selectively regulates promoters of Chlamydia late genes. Mol. Microbiol. 84, 1097–1107. 10.1111/j.1365-2958.2012.08077.x 22624851 PMC3544401

[B171] Rouault T. A. (2006). The role of iron regulatory proteins in mammalian iron homeostasis and disease. Nat. Chem. Biol. 2, 406–415. 10.1038/nchembio807 16850017

[B172] Rouault T. A. Hentze M. W. Caughman S. W. Harford J. B. Klausner R. D. (1988). Binding of a cytosolic protein to the iron-responsive element of human ferritin messenger, R. N. A. Science 241, 1207–1210. 10.1126/science.3413484 3413484

[B173] Rudolph G. Semini G. Hauser F. Lindemann A. Friberg M. Hennecke H.. (2006). The iron control element, acting in positive and negative control of iron-regulated *Bradyrhizobium japonicum* genes, is a target for the Irr protein. J. Bacteriol. 188, 733–744. 10.1128/JB.188.2.733-744.2006 16385063 PMC1347296

[B174] Runyen-Janecky L. J. Payne S. M. (2002). Identification of chromosomal *Shigella flexneri* genes induced by the eukaryotic intracellular environment. Infect. Immun. 70, 4379–4388. 10.1128/IAI.70.8.4379-4388.2002 12117948 PMC128171

[B175] Runyen-Janecky L. J. Reeves S. A. Gonzales E. G. Payne S. M. (2003). Contribution of the *Shigella flexneri* Sit, Iuc, and Feo iron acquisition systems to iron acquisition *in vitro* and in cultured cells. Infect. Immun. 71, 1919–1928. 10.1128/IAI.71.4.1919-1928.2003 12654809 PMC152062

[B176] Sangwan I. Small S. K. O'Brian M. R. (2008). The *Bradyrhizobium japonicum* Irr protein is a transcriptional repressor with high-affinity DNA-binding activity. J. Bacteriol. 190, 5172–5177. 10.1128/JB.00495-08 18539736 PMC2493276

[B177] Schaumburg C. S. Tan M. (2006). Arginine-dependent gene regulation via the argr repressor is species specific in Chlamydia. J. Bacteriol. 188, 919–927. 10.1128/JB.188.3.919-927.2006 16428395 PMC1347356

[B178] Schmitt M. P. (2002). Analysis of a DtxR-Like Metalloregulatory protein, MntR, from *Corynebacterium diphtheriae* that controls expression of an ABC metal transporter by an Mn^2+^-dependent mechanism. J. Bacteriol. 184, 6882–6892. 10.1128/JB.184.24.6882-6892.2002 12446639 PMC135481

[B179] Schmitt M. P. Holmes R. K. (1991). Iron-dependent regulation of diphtheria toxin and siderophore expression by the cloned *Corynebacterium diphtheriae* repressor gene dtxR in C. diphtheriae C7 strains. Infect. Immun. 59, 1899–1904. 1828057 10.1128/iai.59.6.1899-1904.1991 PMC257940

[B180] Schmitt M. P. Holmes R. K. (1994). Cloning, sequence, and footprint analysis of two promoter/operators from corynebacterium diphtheriae that are regulated by the diphtheria toxin repressor (DtxR) and iron. J. Bacteriol. 176, 1141–1149. 10.1128/jb.176.4.1141-1149.1994 8106325 PMC205166

[B181] Schmitt M. P. Twiddy E. M. Holmes R. K. (1992). Purification and characterization of the diphtheria toxin repressor. Proc. Natl. Acad. Sci. U.S.A. 89, 7576–7580. 10.1073/pnas.89.16.7576 1502169 PMC49753

[B182] Scidmore M. A. Fischer E. R. Hackstadt T. (1996). Sphingolipids and glycoproteins are differentially trafficked to the *Chlamydia trachomatis* inclusion. J. Cell Biol. 134, 363–374. 10.1083/jcb.134.2.363 8707822 PMC2120880

[B183] Small S. K. Puri S. Sangwan I. Brian M. R. (2009). Positive control of ferric siderophore receptor gene expression by the irr protein in *Bradyrhizobium japonicum*. J. Bacteriol. 191, 1361–1368. 10.1128/JB.01571-08 19114488 PMC2648218

[B184] Soares M. P. Weiss G. (2015). The Iron age of host—microbe interactions. EMBO Rep. 16, 1–19. 10.15252/embr.201540558 26474900 PMC4641501

[B185] Sönnichsen B. Renzis S. De Nielsen E. Rietdorf J. Zerial M. (2000). Distinct membrane domains on endosomes in the recycling pathway visualized by multicolor imaging of Rab4, Rab5, and Rab11. J. Cell Biol. 149, 901–913. 10.1083/jcb.149.4.901 10811830 PMC2174575

[B186] Spiering M. M. Ringe D. Murphy J. R. Marletta M. A. (2003). Metal stoichiometry and functional studies of the diphtheria toxin repressor. Proc. Natl. Acad. Sci. U.S.A. 100, 1–6. 10.1073/pnas.0737977100 12655054 PMC153003

[B187] Stephens R. S. Kalman S. Lammel C. Fan J. Marathe R. Aravind L.. (1998). Genome sequence of an obligate intracellular pathogen of humans: *Chlamydia trachomatis*. Science 282, 754–759. 10.1126/science.282.5389.754 9784136

[B188] Stojiljkovic I. Hantke K. (1992). Hemin uptake system of *Yersinia enterocolitica*: similarities with other TonB-dependent systems in gram-negative bacteria. EMBO J. 1, 4359–4367. 10.1002/j.1460-2075.1992.tb05535.x PMC557009 1425573

[B189] Sullivan J. L. Weinberg E. D. (1999). Iron and the role of *Chlamydia pneumoniae* in heart disease. Emerging Infect. Dis. 5, 724–726. 10.3201/eid0505.990519 10511533 PMC2627724

[B190] Tailleux L. Neyrolles O. Honore-Bouakline S. Perret E. Sanchez F. Abastado J.-P.. (2003). Constrained intracellular survival of *Mycobacterium tuberculosis* in human dendritic cells. J. Immunol. 170, 1939–1948. 10.4049/jimmunol.170.4.1939 12574362

[B191] Tao X. Murphy J. R. (1992). Binding of the metalloregulatory protein dtxr to the diphtheria operator requires a divalent heavy metal ion and protects the palindromic sequence from DNase. J. Biol. Chem. 267, 21761–21764. 1400485

[B192] Tao X. Boydt J. Murphy J. R. (1992). Specific binding of the diphtheria tox regulatory element DtxR to the tox operator requires divalent heavy metal ions and a 9-base-pair interrupted palindromic sequence. Proc. Natl. Acad. Sci. U.S.A. 89, 5897–5901. 10.1073/pnas.89.13.5897 1631071 PMC49404

[B193] Thomas S. M. Garrity L. F. Brandt C. R. Schobert C. S. Feng G.-S. Taylor M. W.. (1993). IFN-y-mediated antimicrobial response: indoleamine 2,3-dioxygenase-deficient mutant host cells no longer inhibit intracellular *Chlamydia* spp. or toxoplasma growth. J. Immunol. 150, 5529–5534. 8515074

[B194] Thompson C. C. Carabeo R. A. (2011). An optimal method of iron starvation of the obligate intracellular pathogen, *Chlamydia trachomatis*. Front. Microbiol. 2:20. 10.3389/fmicb.2011.00020 21687412 PMC3109288

[B195] Thompson C. C. Nicod S. S. Malcolm D. S. Grieshaber S. S. Carabeo R. A. (2012). Cleavage of a putative metal permease in *Chlamydia trachomatis* yields an iron-dependent transcriptional repressor. Proc. Natl. Acad. Sci. U.S.A. 109, 10546–10551. 10.1073/pnas.1201398109 22689982 PMC3387080

[B196] Thompson J. M. Jones H. A. Perry R. D. (1999). Molecular characterization of the hemin uptake locus (hmu) from *Yersinia pestis* and analysis of hmu mutants for hemin and hemoprotein utilization. Infect. Immun. 67, 3879–3892. 10417152 10.1128/iai.67.8.3879-3892.1999 PMC96668

[B197] Thomson N. R. Holden M. T. G. Carder C. Lennard N. Lockey S. J. Marsh P.. (2008). *Chlamydia trachomatis* : genome sequence analysis of lymphogranuloma venereum isolates. Genome Res. 18, 161–171. 10.1101/gr.7020108 18032721 PMC2134780

[B198] Timms P. Good D. Wan C. Theodoropoulos C. Mukhopadhyay S. Summersgill J. T.. (2009). Differential transcriptional responses between the interferon-γ–induction and iron-limitation models of persistence for *Chlamydia pneumoniae*. J. Microbiol. Immunol. Infect. 42, 27–37. 10.1520/D0850-11.1 19424556

[B199] Toye B. Laferriere C. Claman P. Jessamine P. Peeling R. (1993). Association between antibody to the chlamydial heat-shock protein and tubal infertility. J. Infect. Dis. 168, 1236–1240. 10.1093/infdis/168.5.1236 7901289

[B200] van der Sluijs P. Hull M. Webster P. Mâle P. Goud B. Mellman I. (1992). The small GTP-binding protein rab4 controls an early sorting event on the endocytic pathway. Cell 70, 729–740. 10.1016/0092-8674(92)90307-X 1516131

[B201] van Ooij C. Apodaca G. Engel J. (1997). Characterization of the *Chlamydia trachomatis* vacuole and its interaction with the host endocytic pathway in hela cells. Infect. Immun. 65, 758–766. 9009339 10.1128/iai.65.2.758-766.1997 PMC176122

[B202] Vecerek B. Moll I. Blasi U. (2007). Control of Fur synthesis by the non-coding RNA RyhB and iron-responsive decoding. EMBO J. 26, 965–975. 10.1038/sj.emboj.7601553 17268550 PMC1852835

[B203] Vulpe C. D. Kuo Y. Murphy T. L. Cowley L. Askwith C. Libina N.. (1999). Hephaestin, a ceruloplasmin homologue implicated in intestinal iron transport, is defective in the sla mouse. Nat. Genet. 21, 195–199. 10.1038/5979 9988272

[B204] Wehrl W. Meyer T. F. Jungblut P. R. Müller E. C. Szczepek A. J. (2004). Action and reaction: *Chlamydophila pneumoniae* proteome alteration in a persistent infection induced by iron deficiency. Proteomics 4, 2969–2981. 10.1002/pmic.200400917 15378754

[B205] Wilkinson N. Pantopoulos K. (2014). The IRP/IRE system *in vivo*: Insights from mouse models. Front. Pharmacol. 5:176. 10.3389/fphar.2014.00176 25120486 PMC4112806

[B206] Wilson A. C. Tan M. (2002). Functional analysis of the heat shock regulator HrcA of *Chlamydia trachomatis*. J. Bacteriol. 184, 6566–6571. 10.1128/JB.184.23.6566-6571.2002 12426345 PMC135440

[B207] Winter W. Bazydlo L. Harris N. (2014). The molecular biology of human iron metabolism. Lab. Med. 45, 92–102. 10.1309/LMF28S2GIMXNWHMM 24868988

[B208] Winterbourn C. C. (1995). Toxicity of iron and hydrogen peroxide: the fenton reaction. Toxicol. Lett. 82, 969–974. 10.1016/0378-4274(95)03532-X 8597169

[B209] Worst D. J. M. Gerrits M. Vandenbroucke-Grauls C. M. J. E. Kusters J. G. (1998). *Helicobacter pylori ribBA*-mediated riboflavin production is involved in iron acquisition. J. Bacteriol. 180, 1473–1479. 9515916 10.1128/jb.180.6.1473-1479.1998 PMC107047

[B210] Wyllie S. Raulston J. E. (2001). Identifying regulators of transcription in an obligate intracellular pathogen: a metal-dependent repressor in *Chlamydia trachomatis*. Mol. Microbiol. 40, 1027–1036. 10.1046/j.1365-2958.2001.02453.x 11401709

[B211] Wyrick P. B. (2010). *Chlamydia trachomatis* persistence *in vitro*: an overview. J. Infect. Dis. 201, 88–95. 10.1086/652394 20470046 PMC2878585

[B212] Zhu W. Hunt D. J. Richardson A. R. Stojiljkovic I. (2000). Use of heme compounds as iron sources by pathogenic neisseriae requires the product of the hemO gene. J. Bacteriol. 182, 439–447. 10.1128/JB.182.2.439-447.2000 10629191 PMC94294

[B213] Zohn I. E. De Domenico I. Pollock A. Ward D. M. V. Goodman J. F. Liang X.. (2007). The flatiron mutation in mouse ferroportin acts as a dominant negative to cause ferroportin disease. Blood 109, 4174–4180. 10.1182/blood-2007-01-066068 17289807 PMC1885502

